# Impairment in global protein synthesis uncouples UPR gene induction from *HAC1* mRNA splicing in *Saccharomyces cerevisiae*

**DOI:** 10.3389/fmicb.2025.1629132

**Published:** 2025-09-01

**Authors:** Ralph Allen Capistrano Geronimo, Yuki Ishiwata-Kimata, Yutaka Funahashi, Shingo Izawa, Yukio Kimata

**Affiliations:** ^1^Division of Biological Science, Graduate School of Science and Technology, Nara Institute of Science and Technology, Nara, Japan; ^2^Department of Applied Biology, Graduate School of Science and Technology, Kyoto Institute of Technology, Kyoto, Japan

**Keywords:** yeast, stress response, endoplasmic reticulum, ethanol, unfolded protein response

## Abstract

Upon dysfunction of the endoplasmic reticulum (ER), also known as ER stress, eukaryotic cells alter their transcriptomes. This cytoprotective response is called the unfolded protein response (UPR), which is mediated by Ire1 and *HAC1* in the yeast *Saccharomyces cerevisiae*. ER stress induces self-association and activation of the ER-resident transmembrane endoribonuclease Ire1, which catalyzes the splicing of *HAC1* mRNA. It is widely accepted that *HAC1* mRNA is translated into the nuclear transcription factor Hac1, only after being spliced. To investigate the cellular response to ethanol-induced ER stress, here we gradually added ethanol into *S. cerevisiae* cultures until reaching a final concentration of 16%. Unlike conventional ER stressors, such as tunicamycin and dithiothreitol (DTT), the ethanol exposure did not elicit the Ire1- and *HAC1*-dependent UPR gene induction, even though Ire1 was activated and *HAC1*-mRNA was efficiently spliced. Under the ethanol stress condition, global protein synthesis was nearly abolished, and the Hac1 protein level remained low, despite the presence of spliced *HAC1* mRNA. Furthermore, treatment with the translation inhibitor cycloheximide abolished DTT-induced UPR gene induction. As the UPR signaling pathway requires translation of the spliced *HAC1* mRNA, integrity of the translation machinery is deduced to be essential for UPR gene induction. In summary, we demonstrated that impairment of the translation machinery can actually block UPR gene induction under certain stress conditions. We also propose that this represents an advantageous regulatory system that prevents unnecessary gene induction.

## Introduction

The endoplasmic reticulum (ER) is a membrane-enclosed cellular compartment in which secretory and transmembrane proteins are folded and modified to carry cysteine disulfide bonds and sugar chains. Moreover, lipid molecules, including phospholipids, are primarily synthesized on the ER membrane. Dysfunction or functional shortage of the ER—referred to as ER stress—frequently accompanies the accumulation of unfolded proteins in the ER and is detrimental to cells. Upon ER stress, eukaryotic cells commonly change their gene expression profiles. This cytoprotective response is called the unfolded protein response (UPR), the molecular mechanism of which was initially elucidated using the yeast *Saccharomyces cerevisiae* as a model organism (Ishiwata-Kimata and Kimata, 2023). The ER-resident transmembrane protein Ire1 functions as an ER-stress sensor that initiates the UPR. In response to ER stress, Ire1 self-associates and acquires endoribonuclease activity. In many ascomycetous fungi including *S. cerevisiae*, Ire1 mediates splicing of the *HAC1* gene transcript. In *S. cerevisiae*, the unspliced form of *HAC1* mRNA is translationally inactive and functionless, while the spliced form is translated into the active transcription factor Hac1 (Mori et al., 2000; Rüegsegger et al., 2001). Furthermore, unlike in some other fungal species, *HAC1* mRNA is believed to be the sole substrate of Ire1 in *S. cerevisiae* (Niwa et al., 2005).

Because the *HAC1*-mRNA splicing is a rapid and readily detectable phenomenon, it is frequently monitored to assess ER stress levels and Ire1 activation in *S. cerevisiae*. *HAC1* mRNA undergoes extensive splicing when cells are exposed to tunicamycin, an antibiotic that inhibits N-glycosylation, or dithiothreitol (DTT), a chemical that cleaves disulfide bonds. These ER stressors are known to cause the accumulation of misfolded proteins in the ER, directly leading to the activation of Ire1 (Kimata et al., 2007; Gardner and Walter, 2011). Inositol depletion, which likely induces abnormalities in membrane lipid—referred to as lipid-bilayer stress (LBS)—represents another type of ER stress that activates Ire1 through a distinct mechanism (Promlek et al., 2011; Halbleib et al., 2017).

*S. cerevisiae* carries out ethanol fermentation and is widely used in the food industry and bioethanol production. Therefore, it is an important research question how *S. cerevisiae* cells respond to ethanol-induced stress conditions. We previously reported that ER stress is induced when cells are cultured in the presence of 16% ethanol (Miyagawa et al., 2014). Ethanol is thought to activate Ire1 through a combination of ER accumulation of unfolded proteins and LBS (Miyagawa et al., 2014; Navarro-Tapia et al., 2017; Navarro-Tapia et al., 2018; Tran et al., 2018).

Transcriptome analyses have revealed that the expression levels of hundreds of genes are altered by the UPR (Travers et al., 2000; Kimata et al., 2006). Prominent genes induced by Hac1—referred to as UPR target genes—include those encoding factors involved in ER protein folding, modification, and flux. This finding highlights a key role of the UPR in coping with the accumulation of misfolded proteins in the ER. Moreover, expansion of the ER has been reported to mitigate ER stress, and some genes involved in lipid biosynthesis are also induced by the UPR (Schuck et al., 2009). However, many genes with other or unknown functions are also upregulated (or downregulated) in response to UPR activation.

Given the wide diversity of ER stress stimuli and UPR target genes, in the present study, we investigated whether the UPR signaling pathway produces consistent outcomes when cells are exposed to different types of stress. Unexpectedly, unlike conventional ER stressors, such as tunicamycin and DTT, ethanol stress did not trigger UPR-dependent transcriptome changes, even though Ire1 was activated to induce *HAC1* mRNA splicing. Based on our findings presented here, we propose that UPR activity is modulated by global protein synthesis in *S. cerevisiae* cells.

## Materials and methods

### *S. cerevisiae* strains and plasmids

Transformation of *S. cerevisiae* was performed using the lithium acetate method, as previously described (Adams et al., 1997).

In all experiments other than the fluorescence microscopy, we used the congenic standard strains BY4741 (*MAT**a** his3Δ1 leu2Δ0 met15Δ0 ura3Δ0*) and BY4742 (*MATα his3Δ1 leu2Δ0 lys2Δ0 ura3Δ0*) (Brachmann et al., 1998), along with and their derivatives. The *kanMX4*-based *IRE1*-knockout mutants of BY4741 and BY4742, named Y01907 and Y11907, respectively, were obtained from EUROSCARF.^1^ The plasmid pRS313 is a centromeric low-copy *S. cerevisiae* vector carrying the *HIS3* selectable marker (Sikorski and Hieter, 1989). We previously constructed the plasmid pRS313-IRE1 by inserting the *IRE1* gene (coding region and 5′- and 3′-flanking regions) into pRS313 (Kimata et al., 2004). In this study, we used two pairs of *IRE1 +* and *ire1Δ* strains. One pair consisted of Y11907 transformed with either pRS313-IRE1 or pRS313. The remaining pair comprised BY4741 and Y01907.

To express green fluorescent protein (GFP)-tagged Ire1 under control of the *TEF1* promoter, we used our laboratory strain W303-ire1Δ[pRS313-TEF1p-IRE1-GFP] (*MAT**a** leu2-3,112 trp1-1 can1-100 ura3-1 his3-11,15 ire1: TRP1* [pRS313-TEF1p-IRE1-GFP]) (Ishiwata-Kimata et al., 2013; Le et al., 2016). We did not use BY4741, 4,742, or their derivatives for fluorescence microscopy because they seemed to emit somewhat stronger autofluorescence than the W303-based strains.

The plasmid pCM189 is a centromeric *S. cerevisiae* vector used for the Tet-off system (Garí et al., 1997). We previously inserted the Hac1-coding sequence downstream of the doxycycline-inducible promoter in pCM189 to construct the plasmid pCM189-Hac1 (Ishiwata-Kimata et al., 2025).

The plasmid pML104 is a 2 *μ*-based multicopy *S. cerevisiae* vector used for CRISPR-Cas9 genome editing (Laughery et al., 2015). We modified this plasmid to insert a guide sequence targeting the *HAC1* gene. The following is a partial sequence of the resulting plasmid pML104-HAC1:

gcagtgaaagataaatgatcTACGACAACAACCGCCACTAGTTTTAGAGCTAGaaatagcaagttaaaataag. The sequences derived from pML104 are shown in lowercase letters. The inserted sequence is in uppercase, and the guide sequence targeting *HAC1* is indicated in boldface.

The *HAC1* gene was genome-edited by transforming BY4741 with pML104-HAC1 and donor DNA synthesized by Eurofins Genomics (Tokyo, Japan). The resulting strain, YKY-HA-HAC1, carried an in-frame insertion of three tandem copies of the hemagglutinin (3 × HA) tag after the initiation codon of the *HAC1* gene. The donor DNA sequence was as follows:

cacctcaatggacaactcgagaaatgaatacagaaatatgttttttagcgaaattttcctttcttcttgtcttcttgttttatttaaacttccaaggctttaactcagtgtcaaacataacaacctcctcctcccccacctacgacaacaaccgccactatgTACCCATACGATGTTCCTGACTATGCGGGCTATCCGTATGACGTCCCGGACTATG CAGGATCCTATCCATATGACGTTCCAGATTACGCTgaaatgactgattttgaactaactagtaattcgcaatcgaacctagctatccctaccaacttcaagtcgactctgcctccaaggaaaagagccaagacaaaagaggaaaaggaacagcgaaggatcgagcgtattttgagaaacagaagagctgctcaccagagcagagagaaaaaaagactacatctgcagtatctcgagagaaaaztgttctcttttggaaaatttactgaacagcgtcaaccttga. The sequences of the homology arms corresponding to the *HAC1* 5′-flanking and coding regions are shown in lowercase letters. The sequence corresponding to the 3 × HA tag is indicated in uppercase. The initiation codon of *HAC1* is shown in boldface.

The 3 × HA sequence was also inserted into the same position of the Hac1 gene on pCM189-Hac1 to obtain the plasmid pCM189-HA-Hac1, which was used to express the HA-tagged Hac1 protein (HA-Hac1) under the control of the Tet-off promoter.

### *S. cerevisiae* culturing, stress imposition, and viability test

*Saccharomyces cerevisiae* cells were cultured at 30 °C with shaking in synthetic dextrose (SD) medium containing 2% glucose, 0.66% Difco yeast nitrogen base without amino acids (YNB w/o AA; Becton Dickinson, Franklin Lakes, NJ, United States), and appropriate auxotrophic supplements. Optical density of cultures at 600 nm (OD_600_) was measured using a SmartSpec 3,000 spectrophotometer (Bio-Rad Laboratories, Inc., Hercules, CA, United States). The starting OD_600_ of the cultures was approximately 0.2.

Tunicamycin was purchased from Sigma-Aldrich (Merck, Darmstadt, Germany) and prepared as a 2 mg/mL solution in dimethyl sulfoxide solution. Cycloheximide was purchased from Nacalai Tesque (Kyoto, Japan) and was prepared as a 20 mg/mL aqueous solution. DTT was purchased from Tokyo Chemical Industry (Tokyo, Japan) and prepared as a 1 M aqueous solution. Ethanol (99.5%) were purchased from Nacalai Tesque. These reagents were added to cultures during the fast-growing and exponential phase, which were further incubated at 30 °C with shaking before harvest.

Ethanol concentration in the cultures is expressed as volume/volume percentage (v/v). To stepwise increase the ethanol concentration up to 16%, we first added 0.155 mL of ethanol to 5.0 mL of cultures to reach 3% ethanol, followed by a 60-min incubation. Second, we added 0.165 mL of ethanol to reach 6% ethanol, again incubating for 60 min. Third, we added 0.120 mL of ethanol to reach 8% ethanol, again incubating for 60 min. Fourth, we added 0.120 mL of ethanol to reach 10% ethanol, again incubating for 60 min. Fifth, we added 0.120 mL of ethanol to reach 12% ethanol, again incubating for 60 min. Sixth, we added 0.135 mL of ethanol to reach 14% ethanol, again incubating for 60 min. Finally, after addition of 0.135 mL of ethanol, the cultures were incubated for certain durations, followed by various assays.

Based on the Difco manual (Difco Laboratories, 1984), we mixed pure chemicals to prepare YNB w/o AA lacking inositol, which was then used to make SD medium not containing inositol (SD(−inositol) medium). For inositol depletion, cells in the exponential phase grown in SD medium were harvested, washed six times with SD(−inositol) medium, and further cultured at 30 °C in SD(−inositol) medium.

For RNA and protein extraction, cells were harvested from the cultures at the OD_600_ of approximately 1.0.

To assess cell survival, cultures were appropriately diluted in SD medium and spread onto SD agar plates, which were incubated at 30 °C for 3 days prior to colony counting. The survival ratio in the presence of tunicamycin was calculated as the ratio of colony-forming units (CFU) at a given time point to the CFU immediately before tunicamycin addition. Because cells grew even in the presence of tunicamycin, CFU values were normalized to the OD_600_ of the cultures. The survival ratio in the presence of 16% ethanol was calculated as the ratio of the CFU at a given time point to the CFU immediately before the ethanol concentration reached 16%.

### RNA analysis

Total RNA was extracted from *S. cerevisiae* cells using the hot phenol method (Collart and Oliviero, 2001). Prior to mRNA-sequencing (mRNA-seq) analysis, residual DNA was removed from total RNA samples by DNase I treatment, as described in Ishiwata-Kimata et al. (2025). As previously described (Fauzee et al., 2023), the following procedure was performed by GeomeRead Co. Ltd. (Takamatsu, Japan) for the mRNA-seq analysis. First, total RNA samples were subjected to mRNA purification via the poly(A) method using the KAPA mRNA Capture Kit (KAPA Biosystems, Potters Bar, United Kingdom). Next, DNA libraries were prepared from the mRNA samples using the MGIEasy RNA Directional Library Prep Set (MGI Tech, Shenzhen, China). Finally, DNA sequencing was performed on the DNBSEQ-G400S platform using the DNBSEQ-G400RS High-throughput Sequencing Set (MGI Tech; 2 × 150 bp paired-end reads, 1 Gb data/sample). We then processed the raw FASTQ data using the CLC Genomics Workbench (Qiagen, Venlo, Netherlands). Genes with no or marginal expression (i.e., with the minimum transcript per million (TPM) value of 0.00, or the maximum TPM value less than 1.00) were excluded from further analysis.

To assess the expression levels of selected genes, we performed reverse transcription (RT)-quantitative PCR (qPCR). In accordance with the manufacturer’s instruction, cDNA was synthesized from total RNA samples using the poly(T) primer and ReverTra Ace qPCR RT Master Mix with gDNA Remover (Toyobo, Osaka, Japan). As previously described (Fauzee et al., 2023), cDNA samples were subjected to real-time qPCR analysis using the intercalator method. Table 1 lists the oligonucleotide primers used in this assay. The *TAF10* transcript was used as the reference, and the ΔΔCt method was used to calculate relative gene expression levels.

**Table 1:** Oligonucleotide primers used for the RNA analyses.

Usage	Target	Sequence	Direction
RT	Poly(A)	TTTTTTTTTTTTTTTTT	Reverse
qPCR	*KAR2*	TCTGAAGGTGTCTGCCACAG	Forward
		TTAGTGATGGTGATAGATTCGGATT	Reverse
	*ERO1*	TAACAGCAAATCCGGAACG	Forward
		ACCAAATTTGACCAGCTTGC	Reverse
	*JEM1*	CCAAGATAACGGCCTCTCAG	Forward
		GATGTCGTTGGTGTTGTTGC	Reverse
	*SIL1*	AGCCAGGCAATCTAACTTGG	Forward
		TCAAGTGTCTGCTGTCGATAAGT	Reverse
	*HSP104*	AAGGACGACGCTGCTAACAT	Forward
		CACTTGGTTCAGCGACTTCA	Reverse
	*TAF10*	ATATTCCAGGATCAGGTCTTCCGTAGC	Forward
		GTAGTCTTCTCATTCTGTTGATGTTGTTGTTG	Reverse
Competitive PCR	*HAC1*	TACAGGGATTTCCAGAGCACG (1st exon)	Forward
		TGAAGTGATGAAGAAATCATTCAATTC (2nd exon)	Reverse

As in our previous publication (Le and Kimata, 2021), RT-competitive PCR was performed to assess the splicing level of *HAC1* mRNA. Briefly, cDNA was synthesized from total RNA samples using the poly(T)-primed RT reaction and used as the template for competitive PCR, which amplified products of different sizes from unspliced and spliced *HAC1* mRNAs using the same primer set (Table 1). The PCR products were separated by agarose electrophoresis, and the fluorescence band intensity of the ethidium bromide (EtBr)-stained gels was quantified to calculate *HAC1* mRNA splicing efficiency using the following formula: 100 × [band intensity of spliced form/(band intensity of spliced form + band intensity of unspliced form)].

### Western blot analysis of cell lysates

Cells equivalent to an OD_600_ of 3–5 (depending on the experiments but consistent within each experiment) were harvested by centrifugation, resuspended in 100 μL of lysis buffer containing 50 mM Tris-Cl (pH 7.9), 5 mM ethylenediaminetetraacetic acid, 1% Triton X-100, and protease inhibitors (2 mM phenylmethylsulfonyl fluoride, 100 μg/mL leupeptin, 100 μg/mL aprotinin, 20 μg/mL pepstatin A, and 1:100-diluted Calbiochem Protease Inhibitor Cocktail Set III (Merck)), and lysed by vortexing (top speed, 30 s × 6 times) with 100 μL of glass beads (425–600 μm; Sigma-Aldrich). After clarification by centrifugation at 8000 × g for 10 min, cell lysates were developed by standard sodium dodecyl sulfate-polyacrylamide gel electrophoresis (SDS-PAGE), followed by western blot analysis, as described previously (Mai et al., 2018). The primary antibodies used were mouse monoclonal anti-HA antibody (12CA5; Roche, Basel, Switzerland), rabbit anti-yeast BiP antiserum (Kimata et al., 2003), and mouse monoclonal anti-Pgk1 antibody (22C5D8; Abcam, Cambridge, UK).

### Other techniques

GFP fluorescence in cells was visualized using a BZ-9000E microscope (Keyence, Osaka, Japan) equipped with a CFI Plan Apo λ100 × H objective lens (Nikon) and a preset fluorescent filter set (excitation: 470/30 nm; dichroic mirror: 495 nm; emission: 535/25 nm).

To monitor the protein synthesis rate, cells were grown in SD medium supplemented with auxotrophic requirements (50 mg/L uracil, 75 mg/L leucine, 75 mg/ lysine, and 50 mg/L histidine-HCl) and exposed to the stress stimuli as described above. Then, 1 mL of the resulting cultures (OD_600_ approximately 0.2) were transferred to 13 mL test tubes and mixed with [^35^S]-labeled methionine/cysteine (0.65 Mbq; EXPRE^35^S^35^S Protein Labeling Mix, Revvity, Waltham, MA, United States), followed by further incubation for 8 min. These procedures were performed at 30 °C under aerobically shaking conditions. Protein synthesis was halted by adding 1/10 volume of 100% trichloroacetic acid (TCA) to the cultures. The TCA precipitates were sequentially washed with 5% TCA (five times) and acetone (two times), and their radioactivity was measured using liquid scintillation counting. The protein synthesis ratio was calculated as follows: (^35^S radioactivity of the TCA precipitate)/(culture OD_600_).

Polysome profiles were analyzed as previously described (Inada and Aiba, 2005; Uemura et al., 2020; Ando et al., 2023). Cell extracts were subjected to sucrose gradient-based separation using the Gradient Master 107–201 M and Fractionator 152–002 (BioComp Instruments, Tatamagouche, NS, Canada).

Glucose concentration in the medium was measured using an enzymatic method (Enzytec Liqid D-Glucose; Darmstadt, Germany).

### Statistical analysis

Numerical data are presented as the mean ± standard deviation of triplicate independent cultures. For experiments using cells transformed with pRS313-IRE1 or pRS313, three independent transformants of the same genotype were analyzed.

## Results

### Ethanol stress induces *HAC1* mRNA splicing but not the downstream transcriptome shift

**Figure 1:**
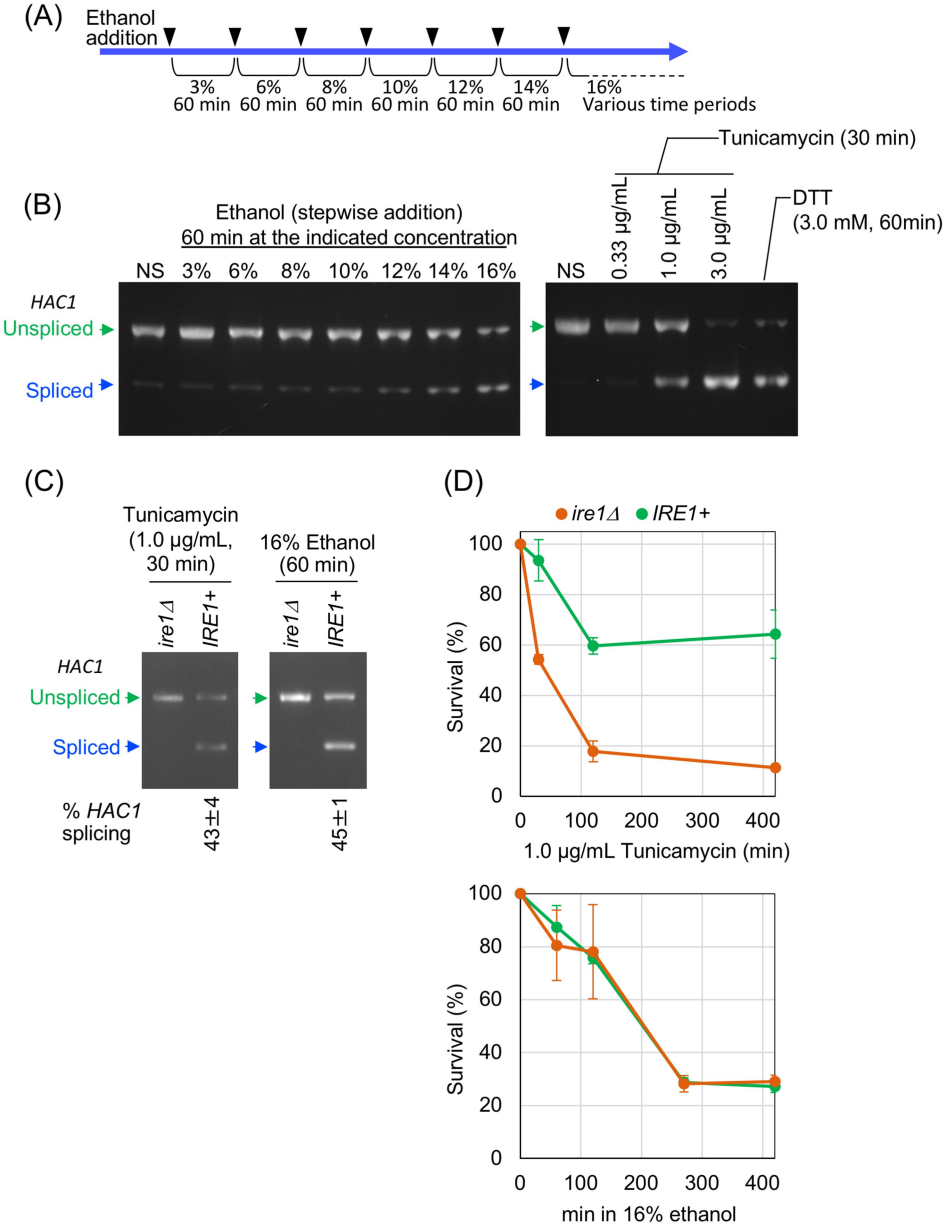
Ire1-dependent *HAC1*-mRNA splicing induced by stepwise increment of the ethanol concentration. **(A)** The procedure for the stepwise addition of ethanol to cultures, reaching a final concentration of 16%, is illustrated. **(B)** *IRE1 +* cells (BY4741) were grown at 30 °C in SD medium and treated with the indicated stress stimuli before extraction of RNA, which was then subjected to RT-competitive PCR to monitor *HAC1* mRNA splicing. The PCR products were separated by electrophoresis on 2% agarose gel, and its EtBr-stained image is shown. **(C,D)** *IRE1 +* cells (Y11907 transformed with pRS313-IRE1) and *ire1Δ* cells (Y11907 transformed with pRS313) were grown at 30 °C in SD medium and treated with tunicamycin or ethanol. Ethanol was added to the cultures using the stepwise addition procedure, and the cells were subsequently incubated in the presence of 16% ethanol for the indicated time periods. The cells were then analyzed for the *HAC1* mRNA splicing, as described in **(B)**, or subjected to a survival assay by colony formation. NS: non-stress.

We previously reported that culturing *S. cerevisiae* cells in the presence of 16% ethanol induces *HAC1* mRNA splicing. In the present study, the ethanol concentration in cultures was increased stepwise, as shown in Figure 1A. Using this stepwise addition method, we observed substantial splicing of *HAC1* mRNA when the ethanol concentration reached 16% (Figure 1B). In addition, we confirmed that DTT and tunicamycin, two widely used ER stressors, also strongly triggered *HAC1*-mRNA splicing (Figure 1B).

It should be noted that, in this study, we increased the ethanol concentration stepwise to avoid acute cell death. When ethanol was added all at once to a final concentration of 16%, cells were rapidly and irreversibly damaged, making it impossible to investigate their physiological responses to ethanol stress (Supplementary Figure S1A). However, when ethanol was added to the cultures using the procedure shown in Figure 1A, cells seemed to lose their viability more slowly (Supplementary Figure S1B).

In response to severe ER stress, Ire1 forms self-associated clusters, which can be visualized as Ire1 puncta, to exert strong *HAC1* mRNA splicing activity (Kimata et al., 2007; Korennykh et al., 2009; Aragón et al., 2009). As shown in Supplementary Figure S2, we examined the intracellular localization of GFP-tagged Ire1 (Ire1-GFP). Consistent our previous observations (Ishiwata-Kimata et al., 2013), Ire1-GFP exhibited a typical double ring-like ER distribution in non-stressed (NS) cells (Supplementary Figure S2A), whereas in cells stressed with tunicamycin or DTT, it appeared clustered at least partly (Supplementary Figures S2B–D). While 2.0 μg/mL tunicamycin apparently caused the punctate distribution of Ire1-GFP (Supplementary Figure S2B), it seemed to partly cluster when tunicamycin was added to the cultures at a lower concentration of 1.0 μg/mL (Supplementary Figure S2C). A similar punctate distribution of Ire1-GFP was observed in cells exposed to 16% ethanol via the stepwise addition method (Supplementary Figure S2E). Therefore, throughout this study, ethanol was added to cultures using the stepwise addition protocol shown in Figure 1A, which sufficiently activated Ire1 to induce *HAC1*-mRNA splicing.

To compare the characteristics of cells carrying and not carrying the *IRE1* gene, we transformed an *IRE1*-knockout strain (Y11907) with either a low-copy *IRE1* plasmid (pRS313-IRE1) or an empty vector (pRS313), and the resulting transformants were used as *IRE1* + and *ire1Δ* strains, respectively. As shown in Figure 1C, *IRE1* + cells exposed to 16% ethanol exhibited a substantial level of *HAC1* mRNA splicing, which was comparable to that induced by 1.0 μg/mL tunicamycin. As expected, the *HAC1* mRNA splicing was not observed in *ire1Δ* cells. In the experiment shown in Figure 1D, cell survival after stress induction was measured using colony formation assay. While tunicamycin impaired the viability of *ire1Δ* cells more severely than that of *IRE1* + cells, no significant difference in viability was observed between *IRE1* + and *ire1Δ* cells under ethanol stress. Therefore, under the ethanol exposure condition employed in this study, the *IRE1*-dependent UPR pathway is unlikely to affect cell survival, although Ire1 is activated to mediate *HAC1*-mRNA splicing.

As shown in Supplementary Figures S3A,B, cell growth was retarded when ethanol was added to cultures, and completely halted when the ethanol concentration reached 16%. We do not deduce that this is due to glucose consumption or diauxic shift, as cells exhibited similar growth patterns upon ethanol exposure even when incubated at different densities (Supplementary Figure S3A). The medium still contained a substantial concentration of glucose even after cells were cultured with 16% ethanol for 60 min (Supplementary Figures S3A,B). Since cells were subjected to various assays at this time point throughout this study, our observations presented in this paper are unlikely to be caused by glucose depletion.

**Figure 2:**
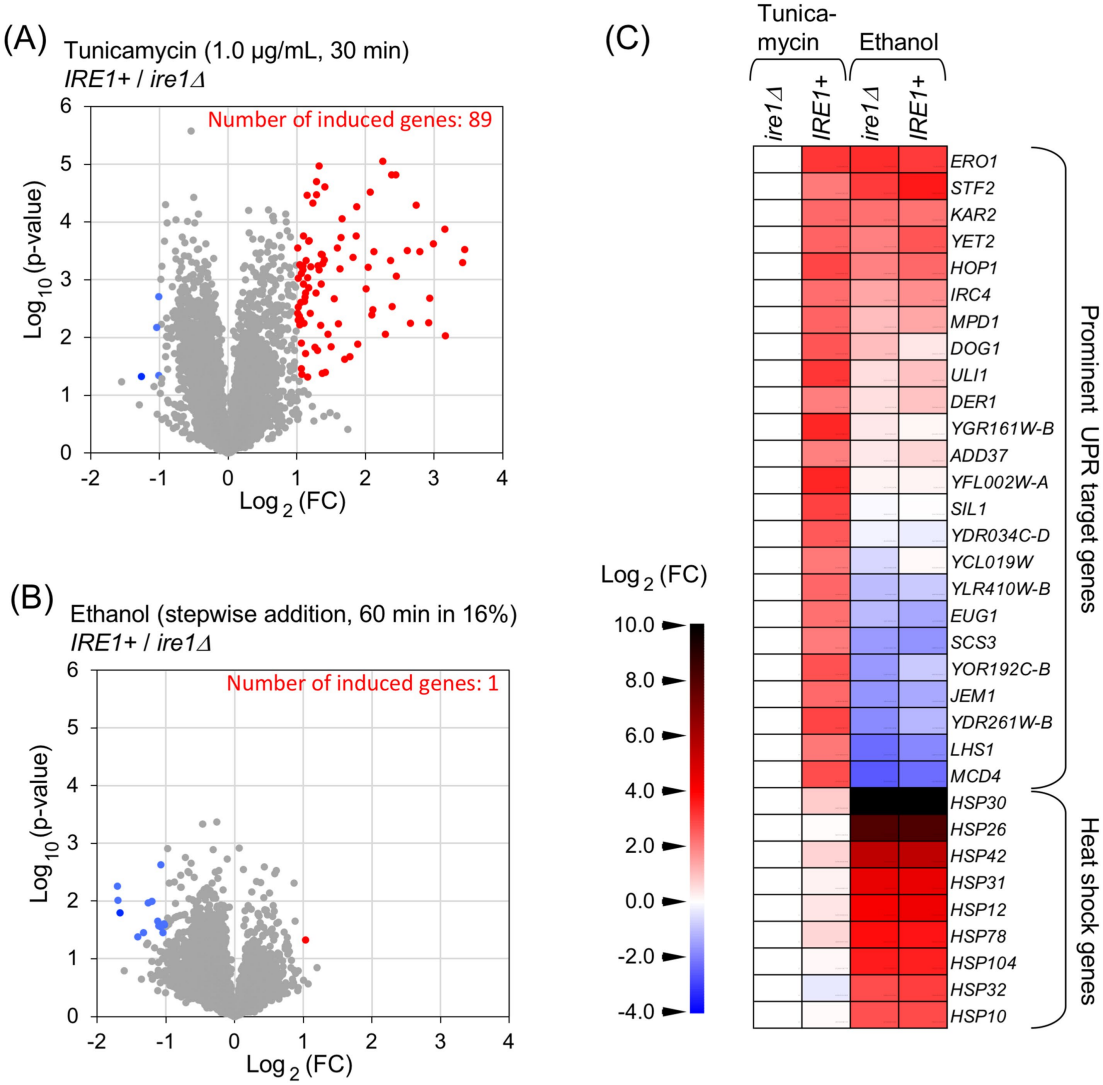
mRNA-seq analysis to investigate the *IRE1*-dependent transcriptome changes. RNA samples taken in the experiments shown in Figure 1C were subjected to mRNA-seq analysis. **(A,B)** Differences in TPM between *IRE1 +* cells and *ire1Δ* cells are presented as volcano plots. Each dot represents a gene. The x-axis represents the Log_2_ of the fold change (FC), and the y-axis represents the negative Log_10_ of the *p*-value. DEGs (*p* < 0.05, FC < –0.5 or >2.0) are indicated by red or blue dots. **(C)** FC of TPM for selected genes relative to tunicamycin-treated *ire1Δ* cells is shown as a heat map. DEGs that were highly expressed in *IRE1 +* cells compared to *ire1Δ* cells (FC > 4.0) under the tunicamycin-treated condition are referred to as “prominent UPR target genes.”

Next, using mRNA-seq, we investigated *IRE1*-dependent transcriptome changes induced by tunicamycin treatment and ethanol exposure. *IRE1* + and *ire1Δ* cells were treated with 1.0 μg/mL tunicamycin for 30 min or exposed to ethanol, which was added stepwise to a final concentration of 16%, followed by an additional incubation for 60 min. Both stimuli induced *HAC1* mRNA splicing at similar levels (Figure 1C). Supplementary Tables S1, S2 present the TPM values obtained from the mRNA-seq analysis. As shown in Figure 2A, a considerable number of genes were differentially expressed between tunicamycin-treated *IRE1* + and *ire1Δ* cells. The change in gene expression was not as pronounced as that presented in our previous study (Ishiwata-Kimata et al., 2025), due to the lower tunicamycin concentration used in this study. In contrast and unexpectedly, almost no difference in gene expression was observed between *IRE1* + and *ire1Δ* cells under the ethanol exposure condition (Figure 2B).

Figure 2C presents a heat map visualizing the mRNA-seq data for selected genes. Supplementary Table S3 lists the numerical data used to generate Figure 2C. The “prominent UPR target genes” refer to differentially expressed genes (DEGs) that were expressed at least fourfold higher in *IRE1* + cells than in *ire1Δ* cells under the tunicamycin treatment condition. For many of these genes, including *SIL1* and *JEM1*, expression levels in ethanol-exposed *IRE1* + and *ire1Δ* cells were similar to those in tunicamycin-treated *ire1Δ* cells, suggesting that they were not induced by the ethanol exposure. However, other genes, such as *ERO1* and *KAR2*, exhibited higher expression in ethanol-exposed *IRE1* + and *ire1Δ* cells than in tunicamycin-treated *ire1Δ* cells. As *ERO1* and *KAR2* are known to be induced not only by the UPR but also by the heat shock response (HSR) (Kohno et al., 1993; Takemori et al., 2006), we examined the expression of representative heat shock protein (HSP) genes and found that they were induced in both *IRE1* + and *ire1Δ* cells upon ethanol exposure (Figure 2C).

To confirm this observation, we used another pair of *IRE1* + and *ire1Δ* strains and monitored expression levels of the representative UPR-target genes, *KAR2*, *ERO1*, *SIL1*, and *JEM1*, using RT-qPCR. BY4741 is a wild-type strain carrying the intact *IRE1* gene on its genome, whereas Y01907 is an *ire1Δ* derivative of BY4741. *KAR2* encodes the ER-resident molecular chaperone BiP, and *SIL1* and *JEM1* encode co-chaperones of BiP that are involved in protein quality control in the ER (Hebert et al., 1995; Nishikawa et al., 2001; Rosam et al., 2018). *ERO1* is involved in protein disulfide bond formation in the ER (Zito, 2015).

**Figure 3:**
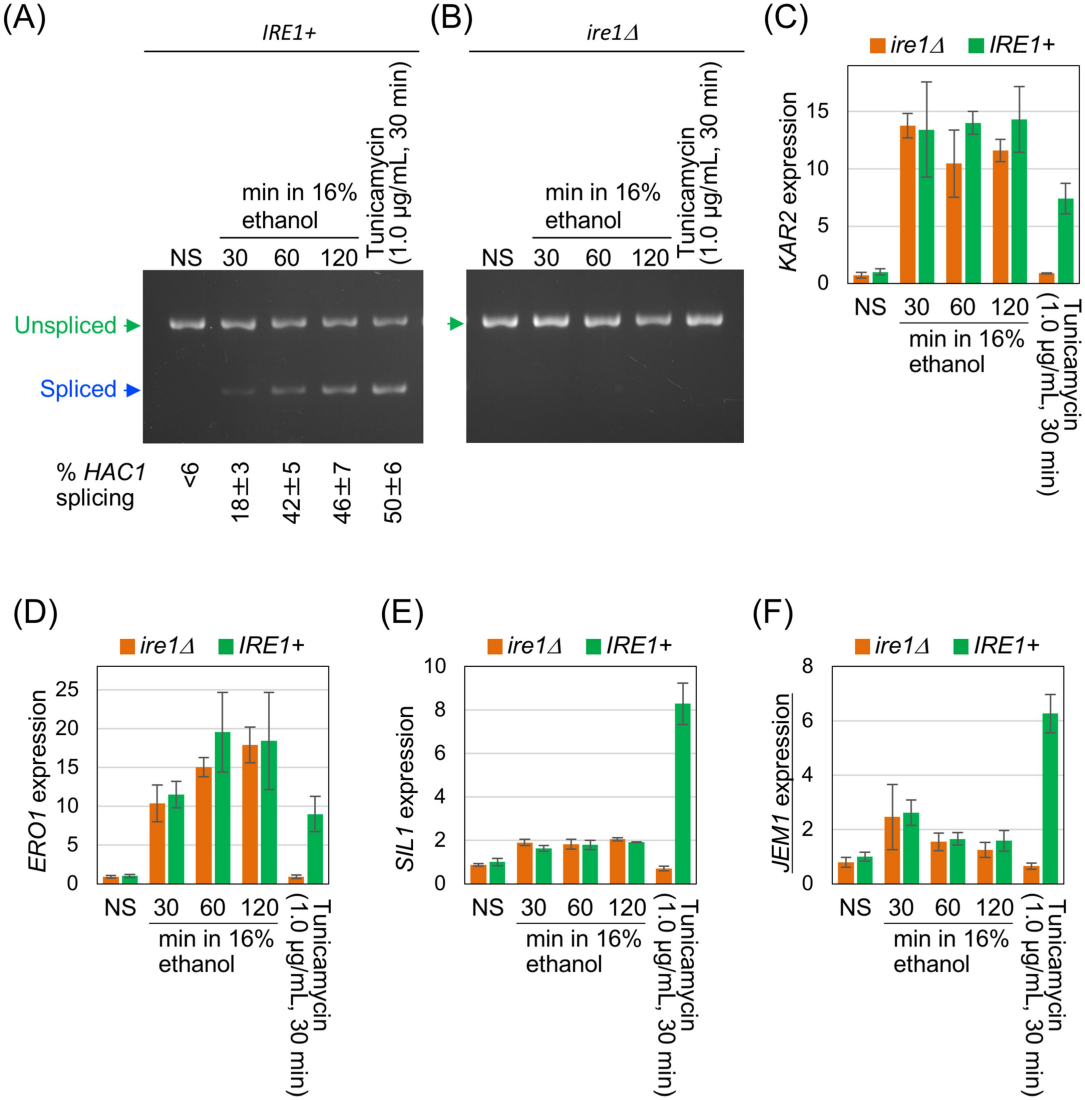
Expression profile of UPR-target genes in cells exposed to ethanol stress. *IRE1 +* cells (BY4741) and *ire1Δ* cells (Y01907) were grown at 30 °C in SD medium and treated with ethanol or tunicamycin. Ethanol was added to the cultures using the stepwise addition method, and the cells were subsequently incubated in the presence of 16% ethanol for the indicated time periods. RNA samples were analyzed by RT-competitive PCR and agarose-gel electrophoresis to assess *HAC1*-mRNA splicing **(A,B)** or by RT-qPCR to measure the relative abundance of selected mRNAs **(C–F)**. The mRNA levels were normalized to that of non-stressed *IRE1 +* cells (set at 1.0) and are presented as the expression levels of the individual gene. NS: non-stress.

In the experiments shown in Figure 3, ethanol was added stepwise to a final concentration of 16%, followed by further incubation for three different time periods. Consistent with the results in Figures 1B,C, ethanol exposure triggered *HAC1*-mRNA splicing in *IRE1 +* cells, but not in *ire1Δ* cells (Figures 3A,B). The cells were also stressed with 1.0 μg/mL tunicamycin for 30 min. As shown in Figures 3C–F, these UPR-target genes were induced by tunicamycin in *IRE1 +* cells but not in *ire1Δ*cells. However, the expression of *KAR2* and *ERO1* was similarly and highly induced in both *IRE1 +* and *ire1Δ* cells by ethanol exposure (Figures 3C,D). We deduce that this is due to the HSR, which does not depend on *IRE1*, rather than the UPR. Other UPR target genes, *SIL1 and JEM1*, were not (or only poorly) induced by ethanol exposure (Figures 3E,F). Taken together, at least under the conditions employed here, ethanol stress activates Ire1 and induces *HAC1* mRNA splicing, but does not trigger downstream gene induction.

The weak and transient induction of *JEM1* upon 30-min exposure of cells to 16% ethanol (Figure 3F, the third and fourth columns) is unlikely to result from the UPR because it was similarly observed in both *IRE1 +* and *ire1Δ* cells. It should also be noted that the *HAC1* mRNA splicing was detectable, but only weak, at this time point (Figure 3A). Since *JEM1* is unlikely to be a heat shock gene, another stress-responsive pathway may be responsible for this weak induction of *JEM1* at this time point.

Supplementary Figure S4 presents the results of the control experiments in which cells were stressed by conventional ER stress stimuli. To induce *HAC1* mRNA splicing for long durations, cells were incubated in the presence of a high concentration (2.5 μg/mL) of tunicamycin, because *HAC1* mRNA splicing subsides when cells are sustainedly treated with 1.0 μg/mL tunicamycin. Cells were also incubated in the presence of two different concentrations of DTT. In addition, cells were cultured under the inositol depletion condition. As shown in Supplementary Figures S4A–D, all of these stress stimuli triggered the *HAC1* mRNA splicing in *IRE1* + cells, but not in *ire1Δ*cells. Supplementary Figure S4E–P shows that these stimuli induced the representative UPR target genes in *IRE1 +* cells but not in *ire1Δ*cells. It should be particularly noted that the UPR target genes were induced by tunicamycin even 420 min after onset of the stress. Therefore, we do not believe that the ethanol exposure failed to induce the UPR target genes simply because the stress exposure time was too long to sustain induction of these genes.

The observations shown in Figure 3 and Supplementary Figure S4 suggest that the HSR, which induces *KAR2* and *ERO1* independently of Ire1, is triggered by ethanol but not by the conventional ER stress stimuli such as tunicamycin, DTT, or inositol depletion. Consistent with this, Supplementary Figure S5 shows that, unlike the conventional ER stress stimuli, ethanol exposure drastically induced *HSP104*, which is a representative heat shock gene.

### Induction of UPR-target genes depends on global protein synthesis

**Figure 4:**
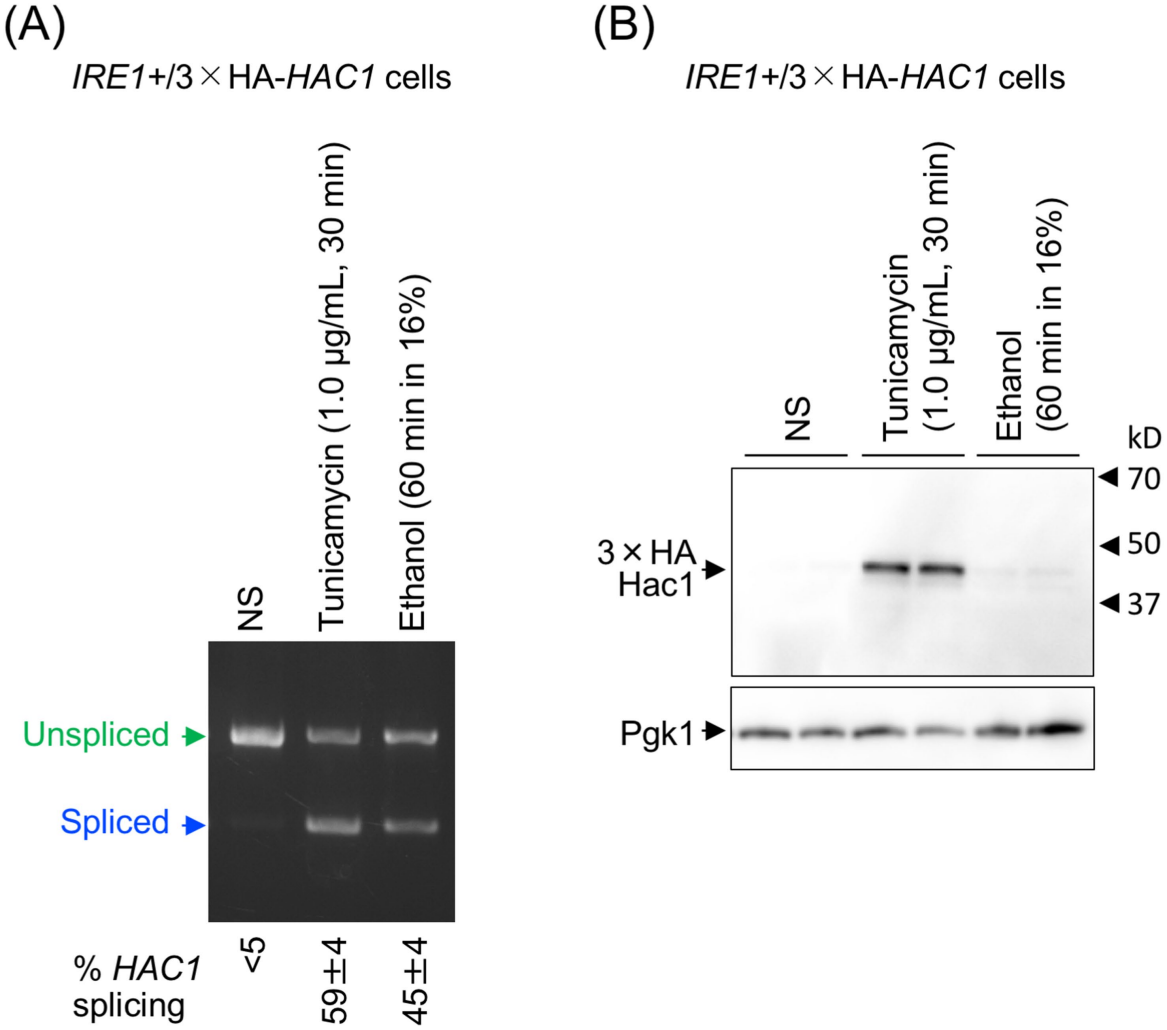
Cellular level of the translation product of *HAC1* mRNA. *IRE1+*/3 × HA-*HAC1* cells (YKY-HA-HAC1) were grown at 30 °C in SD medium and treated with tunicamycin or ethanol. Ethanol was added to the cultures using the stepwise addition method, and the cells were subsequently incubated in the presence of 16% ethanol for 60 min. **(A)** RNA samples were analyzed using RT-competitive PCR and agarose-gel electrophoresis to assess the *HAC1* mRNA splicing. **(B)** Crude cell lysates (equivalent to OD_600_ = 0.4 cells) were run on standard SDS-PAGE and analyzed by anti-HA western blotting. An anti-Pgk1 antibody was used as an endogenous control. NS, non-stress.

Next, we investigated why UPR target genes were not induced by ethanol stress. In the experiment shown in Figure 4, we used *S. cerevisiae* cells carrying the genomic *HAC1* gene with an in-frame insertion of an HA epitope-tag sequence (*IRE1+*/3 × HA-*HAC1* cells). As in the previous experiments, cells were treated with 1.0 μg/mL tunicamycin for 30 min or exposed to ethanol added stepwise to a final concentration of 16%, followed by a further 60-min incubation. These two stress stimuli induced the *HAC1*-mRNA splicing to similar levels (Figure 4A). In the experiment shown in Figure 4B, the HA-tagged Hac1 protein (HA-Hac1) was detected using anti-HA western blotting. While HA-Hac1 was barely detectable in non-stressed (NS) cells, its cellular abundance increased substantially following tunicamycin treatment. This observation is consistent with the well-established view that *HAC1* mRNA is translated only when spliced (Rüegsegger et al., 2001). However, HA-Hac1 protein was not induced upon ethanol exposure (Figure 4B), although *HAC1* mRNA was well spliced (Figure 4A).

**Figure 5:**
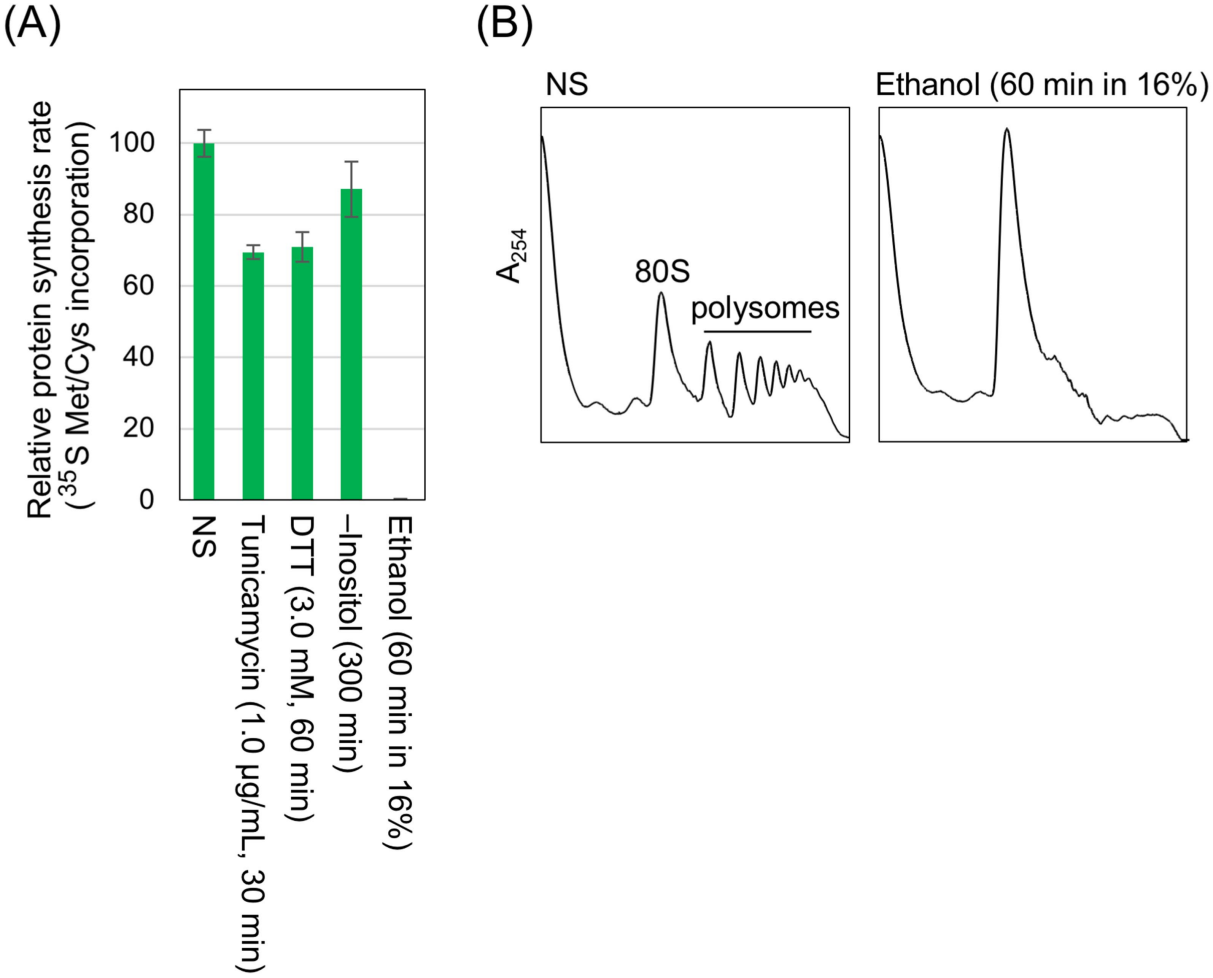
Inhibition of global protein synthesis by ethanol stress but not by conventional ER stress stimuli. **(A)** *IRE1 +* cells (Y11907 transformed with pRS313-IRE1) were grown at 30 °C in SD medium and treated with the indicated stress stimuli. Ethanol was added to the cultures using the stepwise addition method, and the cells were subsequently incubated in the presence of 16% ethanol for 60 min. [^35^S]-labeled Met/Cys was added to the cultures, which were further incubated for 8 min. The cells were then disrupted in the presence of 10% TCA, and the radioactivity in the TCA-insoluble fractions was measured. **(B)** *IRE1 +* cells (BY4741) were grown at 30 °C in SD medium, and ethanol was added (or not added) to the cultures using the stepwise addition method. The cells were then subjected to the polysome profile analysis. NS: non-stress.

In the experiment shown in Figure 5A, cells were stressed as done in other experiments performed throughout this study, and global protein synthesis was assessed by monitoring the incorporation of [^35^S]-labeled methionine/cysteine into TCA-insoluble fractions of cells. Unlike conventional ER stress stimuli such as tunicamycin, DTT, and inositol depletion, the ethanol exposure almost completely abolished [^35^S]-labeled methionine/cysteine incorporation. Consistent with this observation, polysome analysis revealed that polysomal ribosome peaks were lost following the ethanol exposure, indicating the attenuation of global protein synthesis (Figure 5B). Therefore, we presume that under the ethanol exposure condition employed here, cell growth was almost completely halted (Supplementary Figures S3A,B) because protein synthesis was severely suppressed due to the ethanol toxicity. Nevertheless, BiP levels were elevated upon the ethanol exposure (Supplementary Figure S6).

**Figure 6:**
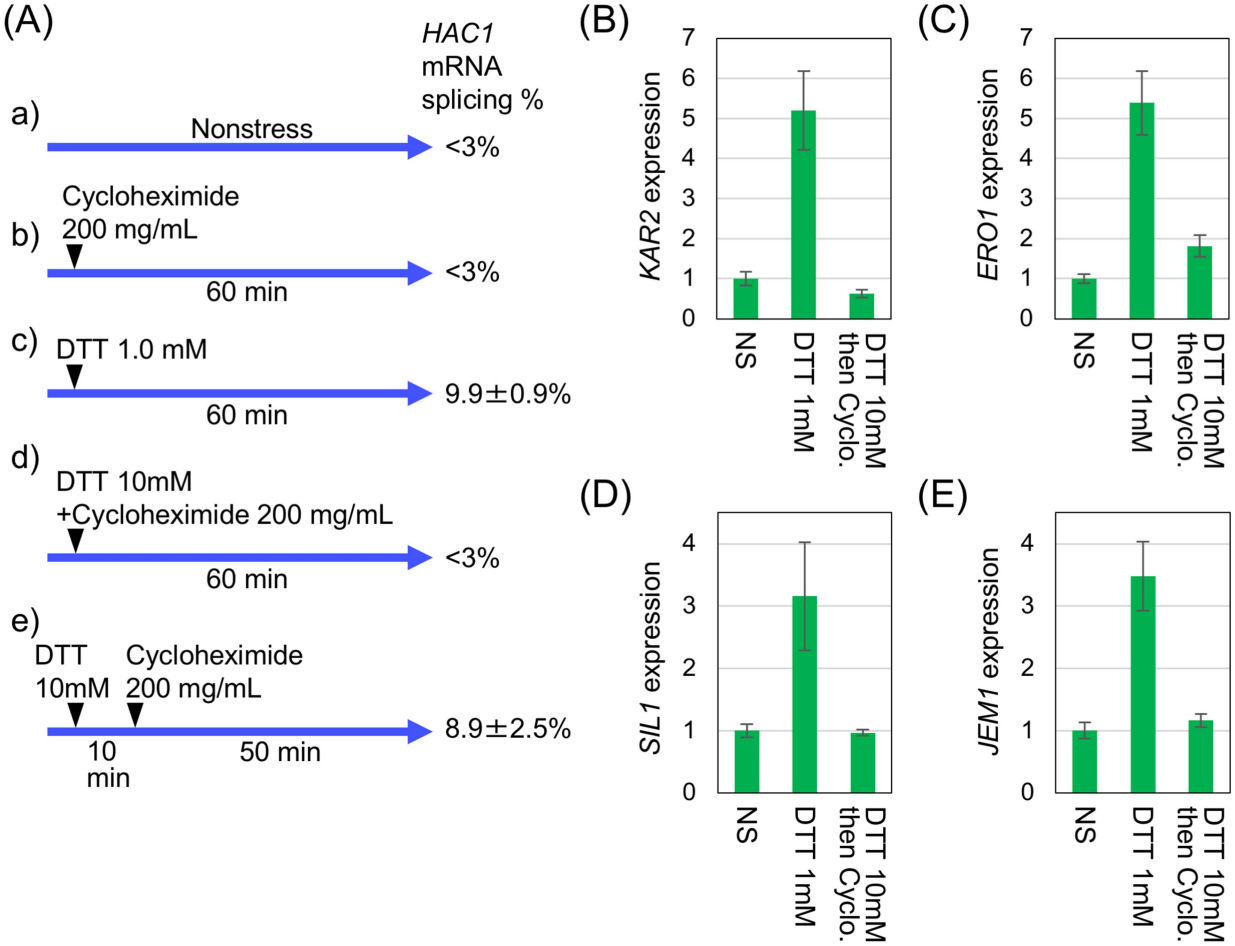
Inhibition of UPR-target-gene induction by cycloheximide. **(A)** *IRE1 +* cells (BY4741) were grown at 30 °C in SD medium and treated with 200 μg/mL cycloheximide and/or the indicated concentrations of DTT, as illustrated. RNA samples were analyzed by RT-competitive PCR and agarose gel electrophoresis to monitor the *HAC1*-mRNA splicing. **(B–E)** RNA samples taken in the experiment shown in (Aa) (leftmost columns), (Ac) (middle columns), and (Ae) (rightmost columns) were analyzed using RT-qPCR to assess the relative abundance of selected mRNAs. The mRNA levels were normalized to that of non-stressed *IRE1 +* cells (set at 1.0) and are presented as the expression levels of the individual gene. NS: non-stress.

Based on these findings, we deduced that under the ethanol stress condition, *HAC1-*mRNA splicing does not lead to downstream gene induction because the spliced form of *HAC1* mRNA is not efficiently translated. To confirm this idea, we investigated whether inhibition of global protein synthesis using another method would yield a similar outcome. In the experiment shown in Figure 6, the protein synthesis inhibitor cycloheximide was added into cultures of *IRE1 +* cells in combination with DTT. Figure 6A shows the treatment conditions and the extent of *HAC1* mRNA splicing. Cycloheximide alone did not induce *HAC1* mRNA splicing (Figure 6Ab). The *HAC1* mRNA splicing was also minimal when DTT and cycloheximide were added simultaneously (Figure 6Ad). However, *HAC1* mRNA was spliced, albeit weakly, when cycloheximide was added into cultures 10 min after 10 mM DTT addition (Figure 6Ae). This sequential treatment induced *HAC1*-mRNA splicing at a level comparable to that observed in cells treated with 1.0 mM DTT alone (Figure 6Ac). Figures 6B–E indicate that the UPR target genes were not induced by the sequential addition of DTT and cycloheximide, despite the splicing of *HAC1* mRNA (Figure 6Ae).

### Artificial induction of the UPR is detrimental under the ethanol stress condition

**Figure 7:**
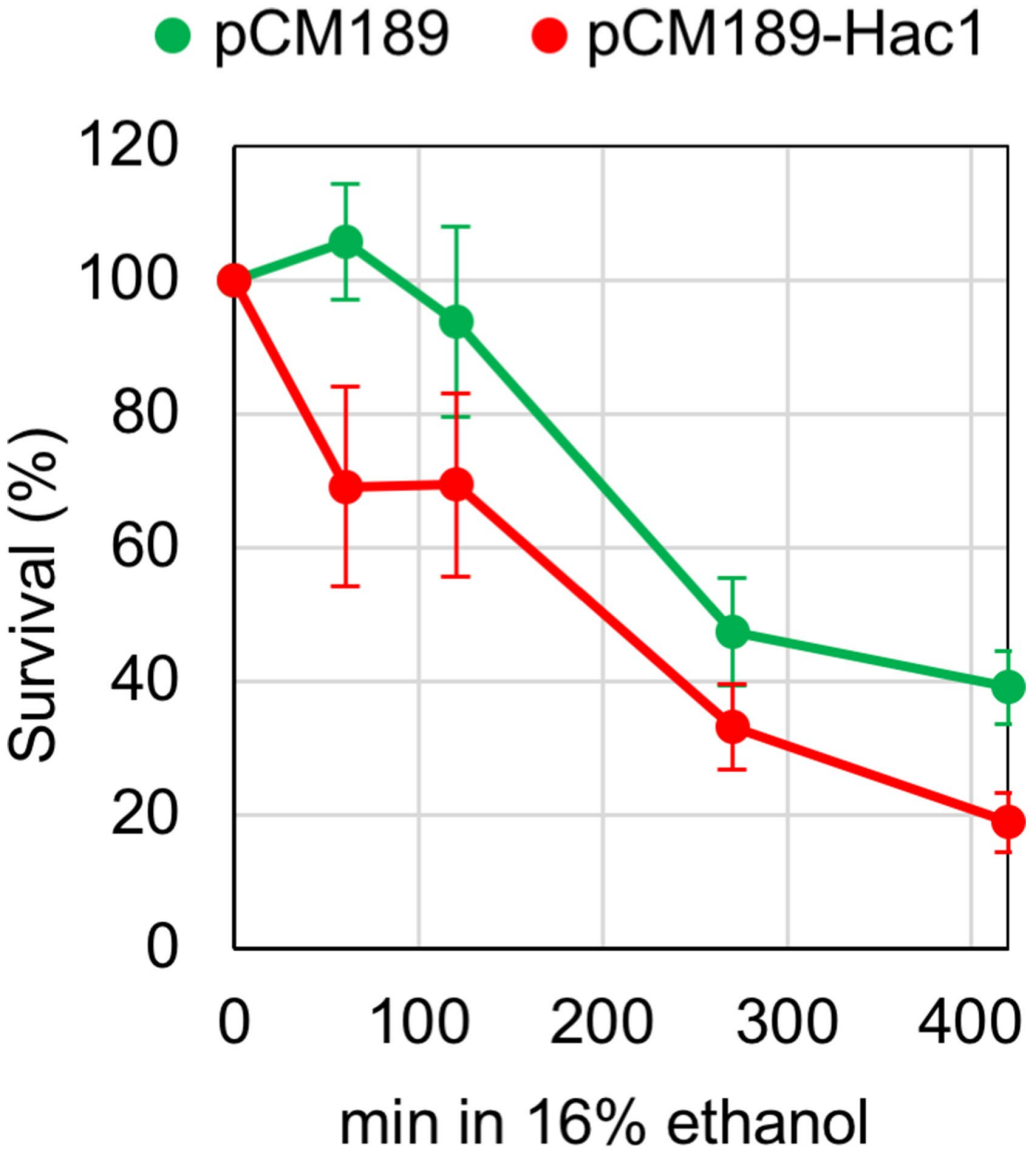
Effect of artificial UPR induction on cellular survival under the ethanol stress condition. *IRE1 +* cells (BY4741) transformed with pCM189-Hac1 or the control empty vector pCM189 were grown at 30 °C in doxycycline-free SD medium. Ethanol was added to the cultures using the stepwise addition method, and cell survival in the presence of 16% ethanol was assessed via colony formation on SD agar plates containing 3.0 μg/mL of doxycycline.

Finally, we examined the physiological significance of our finding that under certain stress conditions, UPR target genes are not induced despite *HAC1*-mRNA splicing. Previously, we constructed a YCp-type plasmid, pCM189-Hac1, for the expression of Hac1 from the intronless *HAC1* mutant gene under the control of the Tet-off promoter in *S. cerevisiae* cells (Ishiwata-Kimata et al., 2025). When cells carrying pCM189-Hac1 were cultured without doxycycline, which represses the Tet-off promoter, Hac1 was expressed, inducing UPR target genes, even under non-stress conditions. The expression level of Hac1 from pCM189-Hac1 was insufficient to affect growth rates under non-stress conditions (Ishiwata-Kimata et al., 2025). In the experiment shown in Figure 7, *IRE1 +* cells carrying either pCM189-Hac1 or the empty vector pCM189 were grown without doxycycline, and ethanol was added stepwise to a final concentration of 16%, followed by a further incubation for monitoring their viability. Cells carrying pCM189-Hac1 exhibited a more rapid loss of viability compared to those carrying pCM189 in the presence of 16% ethanol. Therefore, we assume that cells become highly susceptible to ethanol stress when the UPR is preemptively induced. This observation suggests a negative effect of the UPR and implies the physiological importance of suppressing the UPR under ethanol stress conditions.

The cellular levels of Hac1 under this experimental condition were assessed using the HA epitope-tagging method. Consistent with the result shown in Figure 4B, HA-Hac1 was clearly produced in response to ER stress in cells carrying the genomic HA-*HAC1* gene (Supplementary Figure S7; the leftmost and second lanes). On the contrary, HA-Hac1 was substantially produced from the HA epitope-carrying version of pCM189-Hac1 even under non-stress conditions, and its level decreased but was still detectable for at least 120 min of culturing in the presence of 16% ethanol (Supplementary Figure S7; the third, fourth, and rightmost lanes).

## Discussion

As the 5’-UTR and intron sequences hybridize intramolecularly, the unspliced form of *HAC1* mRNA is not or very poorly translated in *S. cerevisiae* (Rüegsegger et al., 2001). In contrast, the spliced form of *HAC1* mRNA is translated into the active transcription factor Hac1. Therefore, it has been believed that *HAC1* mRNA splicing directly leads to the transcriptional induction of UPR target genes. Based on genome-wide transcriptome analyses conducted by us and others (Travers et al., 2000; Kimata et al., 2006; Ishiwata-Kimata et al., 2025), hundreds of genes have been identified as UPR target genes that are positively regulated by Hac1. In the present study, we confirmed the induction of UPR target genes by DTT, tunicamycin, and inositol depletion, here collectively referred to as conventional ER stress stimuli (Figures 2, 3, and Supplementary Figure S4).

However, in the present study, we demonstrated that splicing of *HAC1* mRNA does not always result in the induction of UPR target genes. Under the conditions employed here, ethanol exposure did not elicit the Ire1- and *HAC1*-dependent induction of UPR target genes, despite efficient splicing of *HAC1* mRNA (Figures 2, 3). It should also be noted that Hac1 was poorly produced under these conditions (Figure 4).

Ethanol stress likely causes broader cellular damage than conventional ER stress stimuli. Consistent with our previous findings (Izawa et al., 2008; Yoshida et al., 2021), ethanol exposure induced the expression of HSP genes (Figure 2 and Supplementary Figure S5), suggesting disruption of cytosolic and nuclear protein integrity (Masser et al., 2020). Moreover, as previously reported (Yamauchi and Izawa, 2016; Kato et al., 2018; Ando et al., 2023) and confirmed in this study (Figure 5), protein synthesis is severely attenuated when cells are exposed to ethanol. In addition, we also demonstrated that DTT triggers *HAC1*-mRNA splicing but not UPR target-gene induction when cells were incubated in the presence of cycloheximide (Figure 6). Therefore, we deduce that the *HAC1*-mRNA splicing does not lead to the UPR target-gene induction when global protein synthesis is compromised and the spliced *HAC1* mRNA is not translated. In other words, the UPR signaling pathway is sensitive to the overall translational capacity of the cell.

We presume that this represents a biologically meaningful regulatory system that halts the induction of UPR target genes under conditions in which global protein synthesis is impaired. As many of the UPR target genes are known to function in ER protein folding, modification, and flux (Travers et al., 2000; Kimata et al., 2006), the UPR can be considered a cellular response to cope with newly synthesized peptides transported into the ER. Therefore, it is plausible that the UPR is dispensable and is turned off even under ER stress when the production of new proteins is severely limited. In line with this, we did not observe a significant difference in cell viability between *IRE1 +* and *ire1Δ* cells under the ethanol exposure (Figure 1D). When inappropriately and highly induced, the UPR can harm cells (Rubio et al., 2011; Chawla et al., 2011), possibly due to the burden of producing unnecessary mRNAs and proteins. In the present study, we demonstrated that cells were highly susceptible to ethanol exposure when Hac1 was expressed prior to stress induction (Figure 7). This observation supports our proposition that UPR induction is unfavorable for cells under ethanol stress.

According to recent publications (Matsuki et al., 2020; Sato et al., 2025), monoubiquitination of ribosomal protein S7 facilitates translation of the spliced *HAC1* mRNA. This observation raises a possibility that the translation of spliced *HAC1* mRNA is regulated in a *HAC1* mRNA-specific manner. However, in the case of ethanol exposure, we deduce that Hac1 is not produced from the spliced *HAC1* mRNA simply because global protein synthesis is severely impaired. Consequently, it may not be necessary to postulate a sophisticated and unique mechanism to explain why Hac1 is not produced under the ethanol exposure condition.

Protein stability and degradation of the translation products of *HAC1* are also important and intriguing topics. As shown in Figure 4, unspliced *HAC1* mRNA is unlikely to produce a translation product, partly because it is rapidly degraded by the proteasome (Di Santo et al., 2016). Moreover, translation of unspliced *HAC1* mRNA is repressed by intramolecular hybridization between its 5’-UTR and intron sequences (Rüegsegger et al., 2001). In contrast, Hac1, the translation product of spliced *HAC1* mRNA, was well detectable in this study when global protein synthesis was not impaired (Figure 4). However, according to Pal et al. (2007), Hac1 is not a stable protein, and is subjected to Ubc3/Cdc4-dependent ubiquitylation for proteasomal degradation. Consequently, the UPR gene expression was completely suppressed when cells were sequentially exposed to DTT and cycloheximide and further incubated for 50 min (Figure 6). On the other hand, when Hac1 was produced prior to stress induction, it was degraded but was still detectable upon the ethanol exposure (Supplementary Figure S7). Therefore, at least under this condition, degradation of Hac1 is unlikely to be sufficient to suppress the UPR gene induction.

In addition to the UPR element recognized by Hac1, *KAR2* and *ERO1* contain the heat shock element (HSE), which is responsible for gene induction upon HSR, on their 5’-UTRs (Kohno et al., 1993; Takemori et al., 2006). Unlike other stress stimuli, ethanol appears to trigger HSE-dependent gene induction even when global protein synthesis is compromised (Tye and Churchman, 2021). As shown in Figures 2, 3, some (but not all) of the UPR target genes, including *KAR2* and *ERO1*, are likely induced via the HSR rather than the UPR under ethanol stress. This may represent a compensatory regulatory mechanism in which certain genes involved in ER proteostasis are induced to alleviate ER stress, even when UPR-dependent gene induction is impaired. In agreement with this idea, Liu and Chang (2008) reported that ER stress is partly alleviated by the HSR in UPR-deficient cells carrying an *ire1Δ* mutation.

We previously proposed that certain genes are selectively translated in *S. cerevisiae* when global protein synthesis is inhibited by ethanol exposure (Yamauchi and Izawa, 2016; Ishikawa et al., 2022). We speculate that such genes may include UPR target genes induced by the HSR. As shown in Supplementary Figure S6, the ethanol exposure increased BiP abundance in cells.

It should also be noted that ethanol elicits different outcomes when added into *S. cerevisiae* cultures using different procedures. When ethanol is added abruptly at high concentrations, cells are acutely and fatally damaged (Supplementary Figure S1A). In contrast, cellular damage caused by ethanol is mitigated when cells are pretreated with lower concentrations of ethanol (Yoshida et al., 2021; Ando et al., 2023). Under such conditions, cells are damaged but remain viable, enabling us to investigate the cellular response to ethanol stress. Consequently, in the present study, ethanol was added stepwise to the cultures. We believe that the gradual increase in ethanol concentration partly mimics the conditions of industrial ethanol fermentation, where ethanol is gradually produced to a maximum concentration of approximately 16%. However, further studies are required to explore what actually occurs during industrial ethanol fermentation. It is also likely that under other conditions of ethanol exposure, the UPR contributes to the downstream gene induction and the mitigation of cellular damage. We previously demonstrated that *ire1Δ* cells exhibit worse survival than *IRE1 +* cells when exposed to ethanol under more chronic conditions (Miyagawa et al., 2014).

In ER-stressed metazoan cells, the IRE1 protein promotes splicing of XBP1 mRNA, which is subsequently translated into a transcription factor (Mori, 2009). We presumed that, similar to the case of *S. cerevisiae HAC1* demonstrated in this study, protein synthesis is required for IRE1- and XBP1-dependent transcriptional induction in metazoan cells. However, metazoan cells also possess another ER-stress sensor, PERK, which phosphorylates the *α* subunit of eukaryotic translation initiation factor 2 to inhibit global protein synthesis upon ER stress (Mori, 2009). According to Gonen et al. (2019), PERK attenuates the expression of XBP1-target genes in mouse cells. As a possible mechanism underlying this phenomenon, we hypothesize that the spliced XBP1 mRNA is poorly translated when global protein synthesis is suppressed by PERK.

## Conclusion

**Figure 8:**
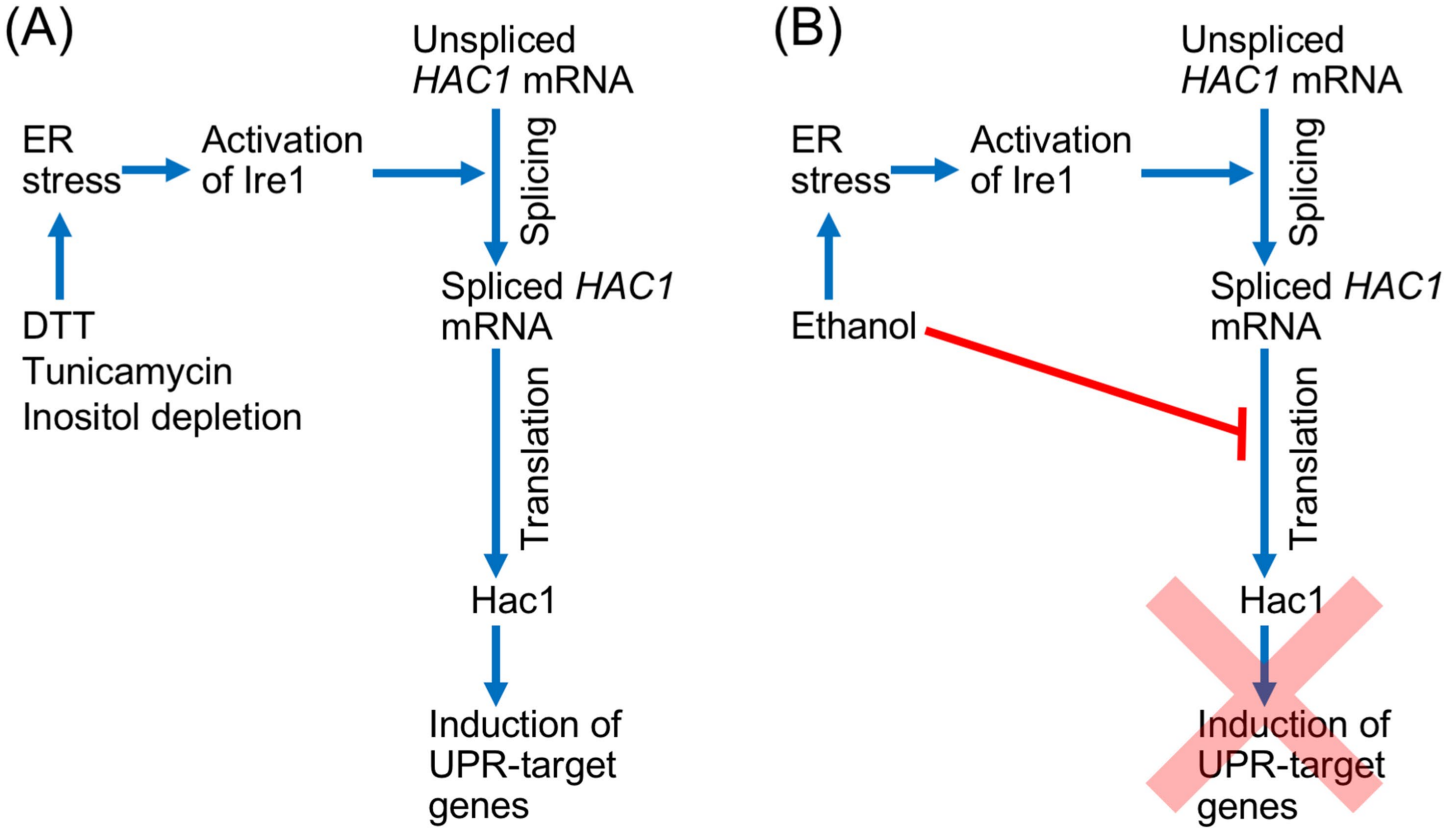
Schematic representation of the UPR signaling pathway and its regulation by global translation capacity in *S. cerevisiae.* **(A)** Conventional ER stress stimuli activate Ire1 and trigger the splicing of *HAC1* mRNA splicing, which straightforwardly yields Hac1 and induces downstream UPR target genes. **(B)** Ethanol induces ER stress, leading to the activation of Ire1 and the splicing of *HAC1* mRNA. However, because ethanol also inhibits global protein synthesis, the spliced *HAC1* mRNA is not translated into Hac1, and the UPR gene induction is abolished.

In conclusion, we propose a novel regulatory model for the UPR in *S. cerevisiae*, as shown in Figure 8. The Ire1- and *HAC1*-dependent UPR signaling pathway includes a protein synthesis step, in which the spliced form of *HAC1* mRNA is translated into Hac1. Therefore, this pathway is dependent on the cellular capacity for protein synthesis. Under conventional ER stress conditions, splicing of *HAC1* mRNA efficiently results in the Hac1 expression and the induction of downstream UPR target genes (Figure 8A). In contrast, when cells are exposed to a stress stimulus that simultaneously damages protein integrity in the ER and cytosol/nucleus, such as ethanol, the splicing of *HAC1* mRNA does not lead to the induction of downstream UPR target genes (Figure 8B). This regulatory mechanism is advantageous for cells, as it prevents unnecessary gene induction. While our primary focus was on the cellular response to ethanol exposure, we also speculate that similar phenomena may occur in response to other stress stimuli that simultaneously impair ER function and global protein synthesis.

We presume that by using this regulatory system, cells can save energy by avoiding the production of unnecessary mRNAs and proteins under harsh stress conditions, in which the energy supply is limited. It is also possible that, by preventing unnecessary gene induction, cells can selectively and effectively cope with stress conditions. This may be particularly important when protein synthesis is severely inhibited, such as during the ethanol exposure. However, it should also be noted that this regulatory system completely abolishes UPR gene induction, even though the expression of certain UPR target genes may contribute to stress mitigation. Possibly to compensate for this limitation, some UPR target genes are upregulated by the HSR in addition to the UPR.

## Data Availability

The original contributions presented in the study are publicly available. This data can be found here: DNA Data Bank of Japan (https://ddbj.nig.ac.jp/search) accession number PRJDB20776.

## Footnotes

^1^ http://www.euroscarf.de/

## References

[ref1] Adams A. Gottschling D. E. Chris A. Kaiser C. A. Stearns T. (1997). Methods in yeast genetics, 1997: A cold Spring Harbor laboratory course manual. Cold Spring Harbor, NY: Cold Spring Harbor Laboratory Press.

[ref2] Ando R. Ishikawa Y. Kamada Y. Izawa S. (2023). Contribution of the yeast bi-chaperone system in the restoration of the RNA helicase Ded1 and translational activity under severe ethanol stress. J. Biol. Chem. 299:105472. doi: 10.1016/j.jbc.2023.105472, PMID: 37979914 PMC10746526

[ref3] Aragón T. van Anken E. Pincus D. Serafimova I. M. Korennykh A. V. Rubio C. A.. (2009). Messenger RNA targeting to endoplasmic reticulum stress signalling sites. Nature 457, 736–740. doi: 10.1038/nature07641, PMID: 19079237 PMC2768538

[ref4] Brachmann C. B. Davies A. Cost G. J. Caputo E. Li J. Hieter P.. (1998). Designer deletion strains derived from *Saccharomyces cerevisiae* S288C: a useful set of strains and plasmids for PCR-mediated gene disruption and other applications. Yeast 14, 115–132. doi: 10.1002/(SICI)1097-0061(19980130)14:2<115::AID-YEA204>3.0.CO;2-2, PMID: 9483801

[ref5] Chawla A. Chakrabarti S. Ghosh G. Niwa M. (2011). Attenuation of yeast UPR is essential for survival and is mediated by *IRE1* kinase. J. Cell Biol. 193, 41–50. doi: 10.1083/jcb.201008071, PMID: 21444691 PMC3082189

[ref6] Collart M. A. Oliviero S. (2001). Preparation of yeast RNA. Curr. Protoc. Mol. Biol. 13:Unit13.12. doi: 10.1002/0471142727.mb1312s23, PMID: 18265096

[ref7] Di Santo R. Aboulhouda S. Weinberg D. E. (2016). The fail-safe mechanism of post-transcriptional silencing of unspliced HAC1 mRNA. eLife 5:e20069. doi: 10.7554/eLife.20069, PMID: 27692069 PMC5114014

[ref8] Difco Laboratories (1984). Difco manual of dehydrated culture media and reagents for microbiology. 10th Edn. Detroit, MI: Difco Laboratories.

[ref9] Fauzee Y. N. B. M. Yoshida Y. Kimata Y. (2023). Endoplasmic stress sensor Ire1 is involved in cytosolic/nuclear protein quality control in. Front. Microbiol. 14:1157146. doi: 10.3389/fmicb.2023.1157146 37415818 PMC10321714

[ref10] Gardner B. M. Walter P. (2011). Unfolded proteins are Ire1-activating ligands that directly induce the unfolded protein response. Science 333, 1891–1894. doi: 10.1126/science.1209126, PMID: 21852455 PMC3202989

[ref11] Garí E. Piedrafita L. Aldea M. Herrero E. (1997). A set of vectors with a tetracycline-regulatable promoter system for modulated gene expression in *Saccharomyces cerevisiae*. Yeast 13, 837–848. doi: 10.1002/(SICI)1097-0061(199707)13:9<837::AID-YEA145>3.0.CO;2-T, PMID: 9234672

[ref12] Gonen N. Sabath N. Burge C. B. Shalgi R. (2019). Widespread PERK-dependent repression of ER targets in response to ER stress. Sci. Rep. 9:4330. doi: 10.1038/s41598-019-38705-5, PMID: 30867432 PMC6416471

[ref13] Halbleib K. Pesek K. Covino R. Hofbauer H. F. Wunnicke D. Hänelt I.. (2017). Activation of the unfolded protein response by lipid bilayer stress. Mol. Cell 67, 673–684.e8. doi: 10.1016/j.molcel.2017.06.012, PMID: 28689662

[ref14] Hebert D. N. Simons J. F. Peterson J. R. Helenius A. (1995). Calnexin, calreticulin, and Bip/Kar2p in protein folding. Cold Spring Harb. Symp. Quant. Biol. 60, 405–415. doi: 10.1101/sqb.1995.060.01.045, PMID: 8824414

[ref15] Inada T. Aiba H. (2005). Translation of aberrant mRNAs lacking a termination codon or with a shortened 3'-UTR is repressed after initiation in yeast. EMBO J. 24, 1584–1595. doi: 10.1038/sj.emboj.7600636, PMID: 15933721 PMC1142571

[ref16] Ishikawa Y. Nishino S. Fukuda S. Nguyet V. T. A. Izawa S. (2022). Severe ethanol stress induces the preferential synthesis of mitochondrial disaggregase Hsp78 and formation of DUMPs in *Saccharomyces cerevisiae*. Biochim. Biophys. Acta Gen. Subj. 1866:130147. doi: 10.1016/j.bbagen.2022.130147, PMID: 35417764

[ref17] Ishiwata-Kimata Y. Kimata Y. (2023). Fundamental and applicative aspects of the unfolded protein response in yeasts. J Fungi 9:989. doi: 10.3390/jof9100989, PMID: 37888245 PMC10608004

[ref18] Ishiwata-Kimata Y. Monguchi M. Geronimo R. A. C. Sugimoto M. Kimata Y. (2025). Artificial induction of the UPR by Tet-off system-dependent expression of Hac1 and its application in *Saccharomyces cerevisiae* cells. Biosci. Biotechnol. Biochem. 89, 562–572. doi: 10.1093/bbb/zbaf006, PMID: 39953902

[ref19] Ishiwata-Kimata Y. Yamamoto Y. H. Takizawa K. Kohno K. Kimata Y. (2013). F-actin and a type-II myosin are required for efficient clustering of the ER stress sensor Ire1. Cell Struct. Funct. 38, 135–143. doi: 10.1247/csf.12033, PMID: 23666407

[ref20] Izawa S. Kita T. Ikeda K. Inoue Y. (2008). Heat shock and ethanol stress provoke distinctly different responses in 3′-processing and nuclear export of HSP mRNA in *Saccharomyces cerevisiae*. Biochem. J. 414, 111–119. doi: 10.1042/BJ20071567, PMID: 18442359

[ref21] Kato S. Yamauchi Y. Izawa S. (2018). Protein synthesis of Btn2 under pronounced translation repression during the process of alcoholic fermentation and wine-making in yeast. Appl. Microbiol. Biotechnol. 102, 9669–9677. doi: 10.1007/s00253-018-9313-x, PMID: 30141081

[ref22] Kimata Y. Ishiwata-Kimata Y. Ito T. Hirata A. Suzuki T. Oikawa D.. (2007). Two regulatory steps of ER-stress sensor Ire1 involving its cluster formation and interaction with unfolded proteins. J. Cell Biol. 179, 75–86. doi: 10.1083/jcb.200704166, PMID: 17923530 PMC2064738

[ref23] Kimata Y. Ishiwata-Kimata Y. Yamada S. Kohno K. (2006). Yeast unfolded protein response pathway regulates expression of genes for anti-oxidative stress and for cell surface proteins. Genes Cells 11, 59–69. doi: 10.1111/j.1365-2443.2005.00921.x, PMID: 16371132

[ref24] Kimata Y. Kimata Y. I. Shimizu Y. Abe H. Farcasanu I. C. Takeuchi M.. (2003). Genetic evidence for a role of BiP/Kar2 that regulates Ire1 in response to accumulation of unfolded proteins. Mol. Biol. Cell 14, 2559–2569. doi: 10.1091/mbc.e02-11-0708, PMID: 12808051 PMC194903

[ref25] Kimata Y. Oikawa D. Shimizu Y. Ishiwata-Kimata Y. Kohno K. (2004). A role for BiP as an adjustor for the endoplasmic reticulum stress-sensing protein Ire1. J. Cell Biol. 167, 445–456. doi: 10.1083/jcb.200405153, PMID: 15520230 PMC2172501

[ref26] Kohno K. Normington K. Sambrook J. Gething M. J. Mori K. (1993). The promoter region of the yeast *KAR2* (BiP) gene contains a regulatory domain that responds to the presence of unfolded proteins in the endoplasmic reticulum. Mol. Cell. Biol. 13, 877–890. doi: 10.1128/mcb.13.2.877-890.1993, PMID: 8423809 PMC358971

[ref27] Korennykh A. V. Egea P. F. Korostelev A. A. Finer-Moore J. Zhang C. Shokat K. M.. (2009). The unfolded protein response signals through high-order assembly of Ire1. Nature 457, 687–693. doi: 10.1038/nature07661, PMID: 19079236 PMC2846394

[ref28] Laughery M. F. Hunter T. Brown A. Hoopes J. Ostbye T. Shumaker T.. (2015). New vectors for simple and streamlined CRISPR-Cas9 genome editing in *Saccharomyces cerevisiae*. Yeast 32, 711–720. doi: 10.1002/yea.3098, PMID: 26305040 PMC4715497

[ref29] Le Q. G. Ishiwata-Kimata Y. Kohno K. Kimata Y. (2016). Cadmium impairs protein folding in the endoplasmic reticulum and induces the unfolded protein response. FEMS Yeast Res. 16:fow049. doi: 10.1093/femsyr/fow049, PMID: 27298227

[ref30] Le Q. G. Kimata Y. (2021). Multiple ways for stress sensing and regulation of the endoplasmic reticulum-stress sensors. Cell Struct. Funct. 46, 37–49. doi: 10.1247/csf.21015, PMID: 33775971 PMC10511038

[ref31] Liu Y. Chang A. (2008). Heat shock response relieves ER stress. EMBO J. 27, 1049–1059. doi: 10.1038/emboj.2008.42, PMID: 18323774 PMC2323268

[ref32] Mai C. T. Le Q. G. Ishiwata-Kimata Y. Takagi H. Kohno K. Kimata Y. (2018). 4-Phenylbutyrate suppresses the unfolded protein response without restoring protein folding in *Saccharomyces cerevisiae*. FEMS Yeast Res. 18:foy016. doi: 10.1093/femsyr/foy016, PMID: 29452364

[ref33] Masser A. E. Ciccarelli M. Andréasson C. (2020). Hsf1 on a leash - controlling the heat shock response by chaperone titration. Exp. Cell Res. 396:112246. doi: 10.1016/j.yexcr.2020.112246, PMID: 32861670

[ref34] Matsuki Y. Matsuo Y. Nakano Y. Iwasaki S. Yoko H. Udagawa T.. (2020). Ribosomal protein S7 ubiquitination during ER stress in yeast is associated with selective mRNA translation and stress outcome. Sci. Rep. 10:19669. doi: 10.1038/s41598-020-76239-3, PMID: 33184379 PMC7661504

[ref35] Miyagawa K. Ishiwata-Kimata Y. Kohno K. Kimata Y. (2014). Ethanol stress impairs protein folding in the endoplasmic reticulum and activates Ire1 in *Saccharomyces cerevisiae*. Biosci. Biotechnol. Biochem. 78, 1389–1391. doi: 10.1080/09168451.2014.921561, PMID: 25130742

[ref36] Mori K. (2009). Signaling pathways in the unfolded protein response: development from yeast to mammals. J. Biochem. 146, 743–750. doi: 10.1093/jb/mvp166, PMID: 19861400

[ref37] Mori K. Ogawa N. Kawahara T. Yanagi H. Yura T. (2000). mRNA splicing-mediated C-terminal replacement of transcription factor Hac1p is required for efficient activation of the unfolded protein response. Proc. Natl. Acad. Sci. USA 97, 4660–4665. doi: 10.1073/pnas.050010197, PMID: 10781071 PMC18289

[ref38] Navarro-Tapia E. Pérez-Torrado R. Querol A. (2017). Ethanol effects involve non-canonical unfolded protein response activation in yeast cells. Front. Microbiol. 8:383. doi: 10.3389/fmicb.2017.00383, PMID: 28326077 PMC5339281

[ref39] Navarro-Tapia E. Querol A. Pérez-Torrado R. (2018). Membrane fluidification by ethanol stress activates unfolded protein response in yeasts. Microb. Biotechnol. 11, 465–475. doi: 10.1111/1751-7915.13032, PMID: 29469174 PMC5902320

[ref40] Nishikawa S. I. Fewell S. W. Kato Y. Brodsky J. L. Endo T. (2001). Molecular chaperones in the yeast endoplasmic reticulum maintain the solubility of proteins for retrotranslocation and degradation. J. Cell Biol. 153, 1061–1070. doi: 10.1083/jcb.153.5.1061, PMID: 11381090 PMC2174341

[ref41] Niwa M. Patil C. K. DeRisi J. Walter P. (2005). Genome-scale approaches for discovering novel nonconventional splicing substrates of the Ire1 nuclease. Genome Biol. 6:R3. doi: 10.1186/gb-2004-6-1-r3, PMID: 15642095 PMC549064

[ref42] Pal B. Chan N. C. Helfenbaum L. Tan K. Tansey W. P. Gething M. J. (2007). SCFCdc4-mediated degradation of the Hac1p transcription factor regulates the unfolded protein response in *Saccharomyces cerevisiae*. Mol. Biol. Cell 18, 426–440. doi: 10.1091/mbc.e06-04-0304, PMID: 17108329 PMC1783797

[ref43] Promlek T. Ishiwata-Kimata Y. Shido M. Sakuramoto M. Kohno K. Kimata Y. (2011). Membrane aberrancy and unfolded proteins activate the endoplasmic reticulum stress sensor Ire1 in different ways. Mol. Biol. Cell 22, 3520–3532. doi: 10.1091/mbc.E11-04-0295, PMID: 21775630 PMC3172275

[ref44] Rosam M. Krader D. Nickels C. Hochmair J. Back K. C. Agam G.. (2018). Bap (Sil1) regulates the molecular chaperone BiP by coupling release of nucleotide and substrate. Nat. Struct. Mol. Biol. 25, 90–100. doi: 10.1038/s41594-017-0012-6, PMID: 29323281

[ref45] Rubio C. Pincus D. Korennykh A. Schuck S. El-Samad H. Walter P. (2011). Homeostatic adaptation to endoplasmic reticulum stress depends on Ire1 kinase activity. J. Cell Biol. 193, 171–184. doi: 10.1083/jcb.201007077, PMID: 21444684 PMC3082176

[ref46] Rüegsegger U. Leber J. H. Walter P. (2001). Block of *HAC1* mRNA translation by long-range base pairing is released by cytoplasmic splicing upon induction of the unfolded protein response. Cell 107, 103–114. doi: 10.1016/s0092-8674(01)00505-0, PMID: 11595189

[ref47] Sato N. Nakano Y. Matsuki Y. Tomomatsu S. Li S. Matsuo Y.. (2025). Crucial roles of Grr1 in splicing and translation of *HAC1* mRNA upon unfolded stress response. Nat. Commun. 16:2172. doi: 10.1038/s41467-025-57360-1, PMID: 40038285 PMC11880305

[ref48] Schuck S. Prinz W. A. Thorn K. S. Voss C. Walter P. (2009). Membrane expansion alleviates endoplasmic reticulum stress independently of the unfolded protein response. J. Cell Biol. 187, 525–536. doi: 10.1083/jcb.200907074, PMID: 19948500 PMC2779237

[ref49] Sikorski R. S. Hieter P. (1989). A system of shuttle vectors and yeast host strains designed for efficient manipulation of DNA in *Saccharomyces cerevisiae*. Genetics 122, 19–27. doi: 10.1093/genetics/122.1.19, PMID: 2659436 PMC1203683

[ref50] Takemori Y. Sakaguchi A. Matsuda S. Mizukami Y. Sakurai H. (2006). Stress-induced transcription of the endoplasmic reticulum oxidoreductin gene *ERO1* in the yeast *Saccharomyces cerevisiae*. Mol. Gen. Genomics. 275, 89–96. doi: 10.1007/s00438-005-0065-9, PMID: 16292667

[ref51] Tran D. M. Takagi H. Kimata Y. (2018). Categorization of endoplasmic reticulum stress as accumulation of unfolded proteins or membrane lipid aberrancy using yeast Ire1 mutants. Biosci. Biotechnol. Biochem. 83, 326–329. doi: 10.1080/09168451.2018.1530098 30319071

[ref52] Travers K. J. Patil C. K. Wodicka L. Lockhart D. J. Weissman J. S. Walter P. (2000). Functional and genomic analyses reveal an essential coordination between the unfolded protein response and ER-associated degradation. Cell 101, 249–258. doi: 10.1016/s0092-8674(00)80835-1, PMID: 10847680

[ref53] Tye B. W. Churchman L. S. (2021). Hsf1 activation by proteotoxic stress requires concurrent protein synthesis. Mol. Biol. Cell 32, 1800–1806. doi: 10.1091/mbc.E21-01-0014, PMID: 34191586 PMC8684711

[ref54] Uemura S. Mochizuki T. Amemiya K. Kurosaka G. Yazawa M. Nakamoto K.. (2020). Amino acid homeostatic control by TORC1 in *Saccharomyces cerevisiae* under high hydrostatic pressure. J. Cell Sci. 133:jcs245555. doi: 10.1242/jcs.245555, PMID: 32801125

[ref55] Yamauchi Y. Izawa S. (2016). Prioritized expression of *BTN2* of *Saccharomyces cerevisiae* under pronounced translation repression induced by severe ethanol stress. Front. Microbiol. 7:1319. doi: 10.3389/fmicb.2016.01319, PMID: 27602028 PMC4993754

[ref56] Yoshida M. Kato S. Fukuda S. Izawa S. (2021). Acquired resistance to severe ethanol stress in *Saccharomyces cerevisiae* protein quality control. Appl. Environ. Microbiol. 87, e02353–e02320. doi: 10.1128/AEM.02353-20 33361368 PMC8105026

[ref57] Zito E. (2015). ERO1: A protein disulfide oxidase and H_2_O_2_ producer. Free Radic. Biol. Med. 83, 299–304. doi: 10.1016/j.freeradbiomed.2015.01.011, PMID: 25651816

