# Supplementary material for: Impairment in global protein synthesis uncouples UPR gene induction from HAC1 mRNA splicing in Saccharomyces cerevisiae
Source: Front Microbiol. 2025 Sep 1;16:1629132. doi: 10.3389/fmicb.2025.1629132 (PMC12435717; doi:10.3389/fmicb.2025.1629132)
Supplement: Supplementary file 1 [file Data_Sheet_1.pdf]

Figure S1

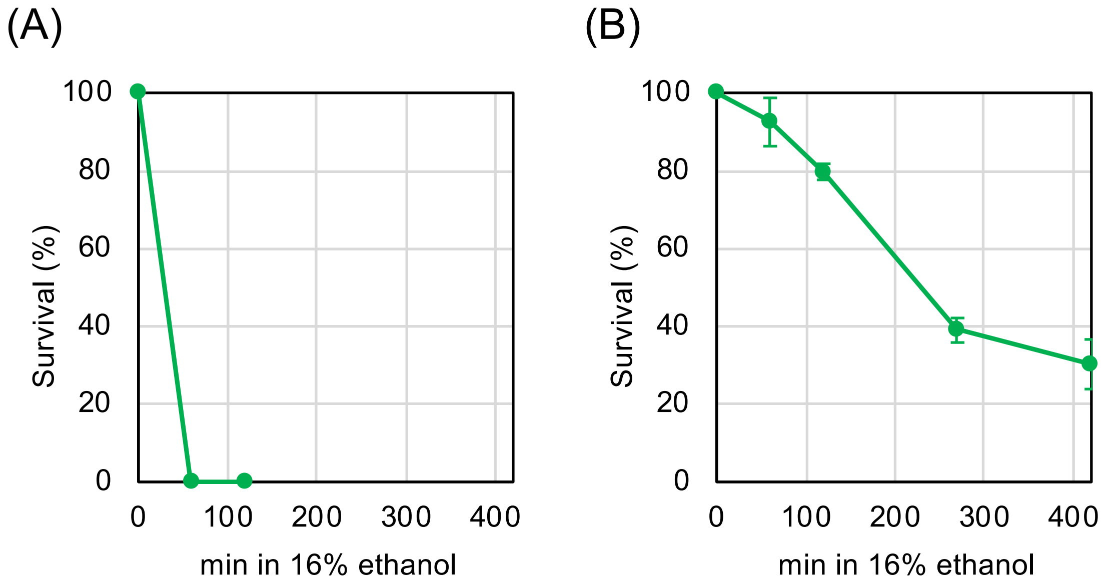

Figure S1 Survival of cells under ethanol stress conditions

IRE1+ cells (BY4741) were grown at 30 ° C in SD medium, and ethanol was added to the cultures to a final concentration of 16% either all at once (A) or using the stepwise addition method (B). Cell survival was then assessed using the colony formation assay.

Figure S2

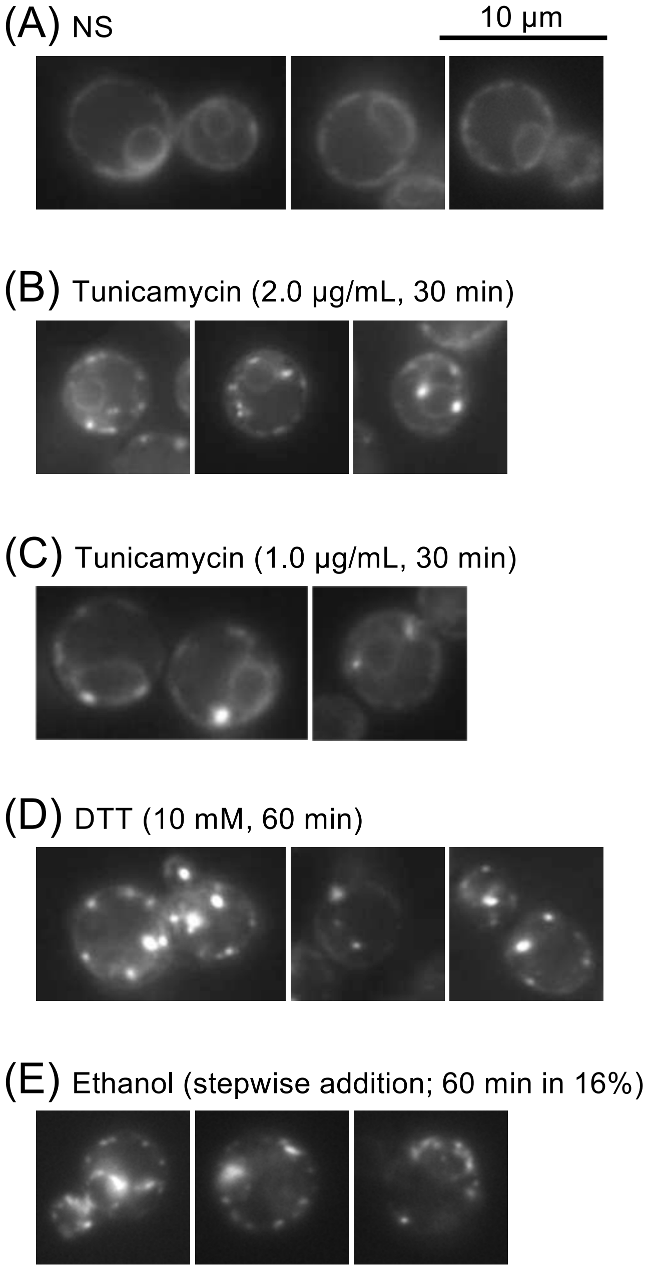

Figure S2 Cluster formation of Ire1-GFP under various stress conditions

Cells expressing Ire1-GFP (Y11907 transformed with pRS313-Ire1-GFP) were grown at 30 ° C in SD medium, (A) The cultures remained non-stressed (NS). (B, C, and D) Cells were treated with tunicamycin or DTT. (E) Ethanol was added to the cultures using the stepwise addition method, and the cells were subsequently incubated in the presence of 16% ethanol for 60 min. The cells were then observed under a fluorescence microscope.

Figure S3

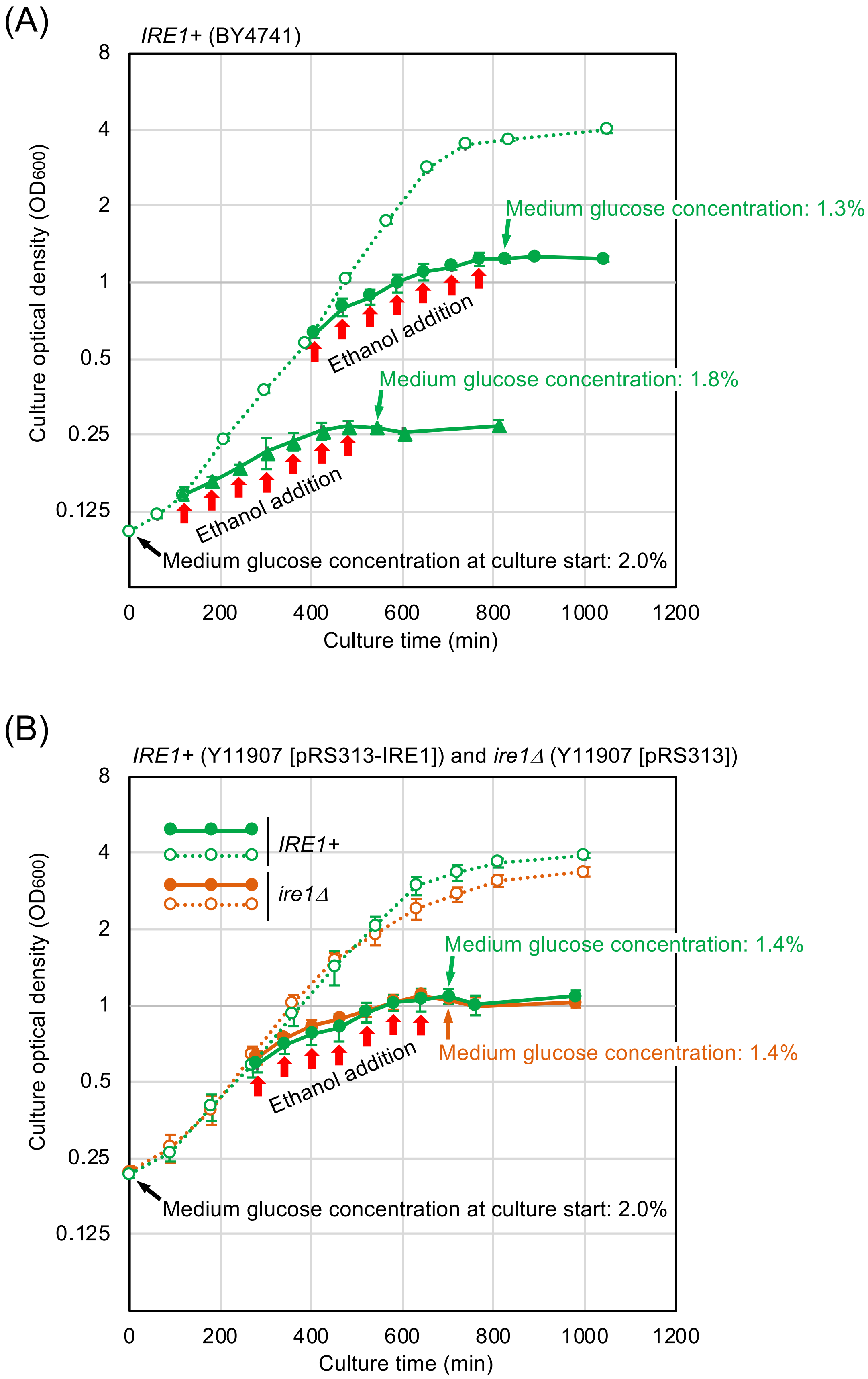

(A)
IRE1+ (BY4741)
8
4
2
1
0.5
0.25
0.125
Culture optical density (OD600)
Medium glucose concentration: 1.3%
Ethanol addition
Medium glucose concentration: 1.8%
Ethanol addition
Medium glucose concentration at culture start: 2.0%
0
200
400
600
800
1000
1200
Culture time (min)
(B)
IRE1+ (Y11907 [pRS313-IRE1]) and ire1Δ (Y11907 [pRS313])
IRE1+
ire1Δ
Medium glucose concentration: 1.4%
Ethanol addition
Medium glucose concentration: 1.4%
Medium glucose concentration at culture start: 2.0%
Culture optical density (OD600)
Culture time (min)

Figure S3 Growth property of cells under ethanol stress and non-stress conditions
To monitor the OD_{600} of cultures, cells were grown at 30 ° C in SD medium under non-stress conditions (dotted lines), or ethanol was added to cultures using the stepwise addition method at the time points indicated by red arrows, followed by further incubation in the presence of 16% ethanol (solid lines). (A) *IRE1+* cells (BY4741) were used in this assay. The onset of ethanol stress (the first addition of ethanol) was at two different time points when OD_{600} of cultures was approximately 0.15 or 0.6. (B) *IRE1+* cells (Y11907 transformed with pRS313-IRE1) and *ire1Δ* cells (Y11907 transformed with pRS313) were used in this assay. The glucose concentration in the medium at a time point (16% ethanol for 60 min) was also measured and is shown in the panels.

Figure S4

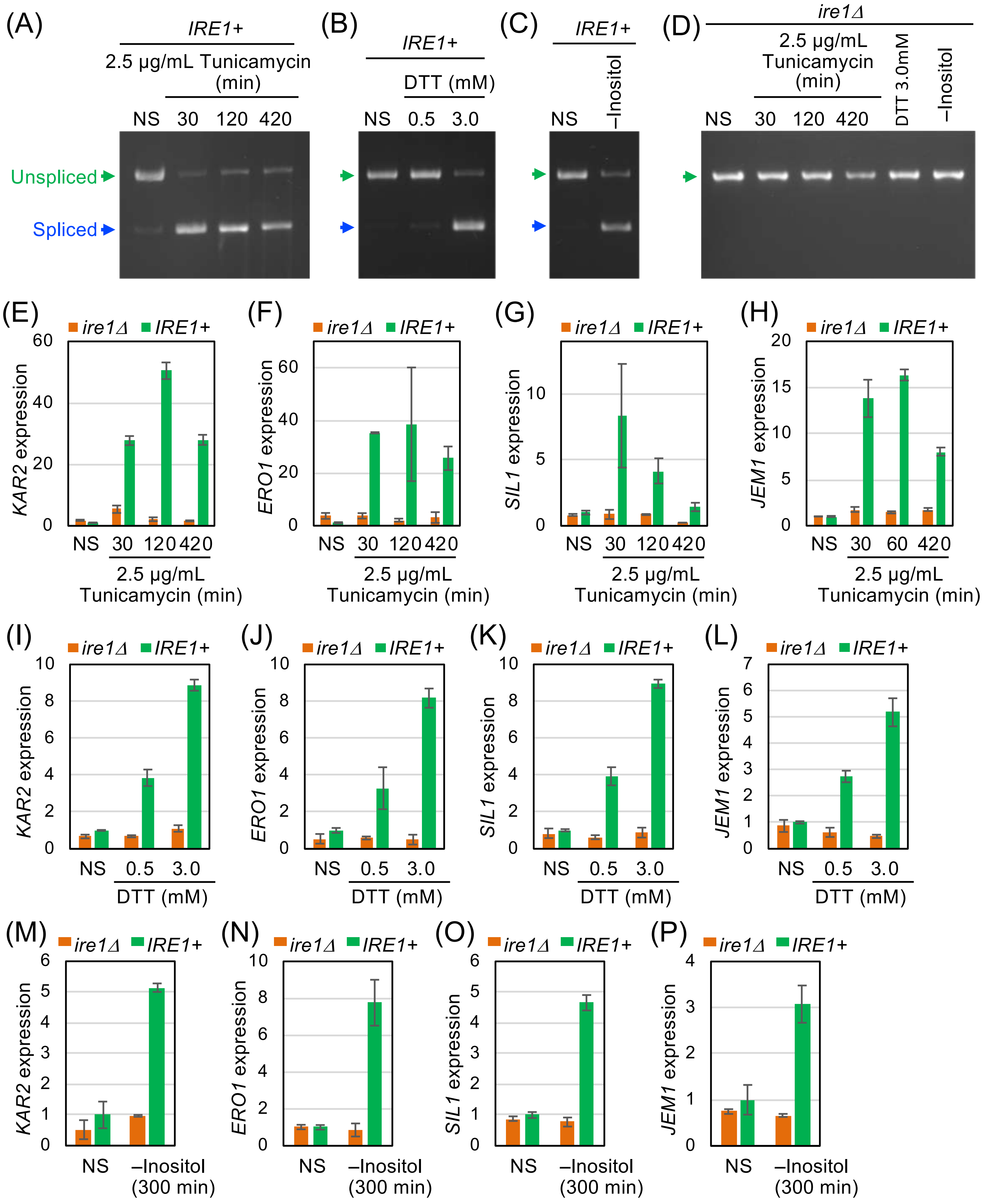

(A)
IRE1+
2.5 μg/mL Tunicamycin (min)
NS 30 120 420
Unspliced
Spliced
(B)
IRE1+
DTT (mM)
NS 0.5 3.0
(C)
IRE1+
NS –Inositol
(D)
ire1Δ
2.5 μg/mL Tunicamycin (min)
NS 30 120 420
DTT 3.0mM
–Inositol
(E)
ire1Δ IRE1+
KAR2 expression
2.5 μg/mL Tunicamycin (min)
(F)
ERO1 expression
(G)
SIL1 expression
(H)
JEM1 expression
NS 30 60 420
(I)
(J)
(K)
(L)
DTT (mM)
(M)
(N)
(O)
(P)
NS –Inositol (300 min)

Figure S4 Expression profile of UPR-target genes in cells exposed to conventional ER stress stimuli

IRE1+ cells (BY4741) and *ire1Δ* cells (Y01907) were grown at 30 ° C in SD medium and exposed to the indicated stress stimuli. For the DTT exposure, the cells were incubated in the presence of DTT for 30 min. RNA samples were analyzed by RT-competitive PCR and agarose gel electrophoresis to assess *HAC1*-mRNA splicing (A–D) or by RT-qPCR to measure the relative abundance of selected mRNAs (E–P). The mRNA levels were normalized to that of non-stressed *IRE1+* cells (set at 1.0) and are presented as the expression levels of the individual gene. NS: non-stress.

Figure S5

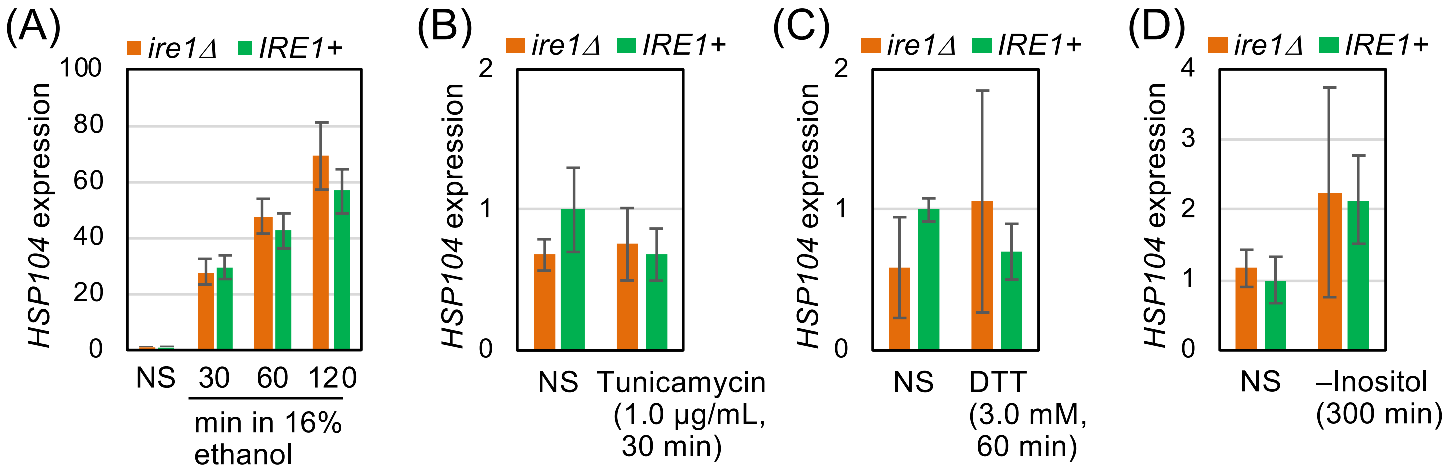

Figure S5 Expression profile of *HSP104* under various stress conditions

IRE1+ cells (BY4741) and *ire1Δ* cells (Y01907) were grown at 30 ° C in SD medium and treated with the indicated stress stimuli. Ethanol was added to the cultures using the stepwise addition method, and the cells were subsequently incubated in the presence of 16% ethanol for the indicated time periods. RNA samples were analyzed using RT-qPCR to monitor the relative abundance of *HSP104* mRNA. The mRNA levels were normalized to that of non-stressed *IRE1+* cells (set at 1.0) and are presented as the *HSP104* expression levels. NS: non-stress.

Figure S6

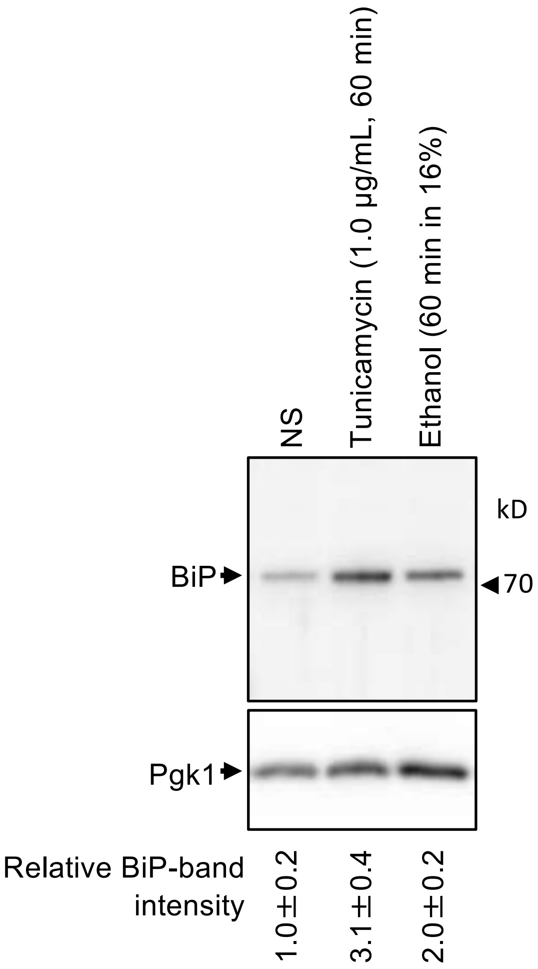

Figure S6 BiP abundance in cells stressed with tunicamycin or ethanol

IRE1+ cells (BY4741) were grown at 30 ° C in SD medium and treated with tunicamycin or ethanol. Ethanol was added to the cultures using the stepwise addition method, and the cells were subsequently incubated in the presence of 16% ethanol for 60 min. Crude cell lysates (equivalent to OD_{600}=0.05 cells) were run on standard SDS-PAGE and analyzed using anti-BiP western blotting. An anti-Pgk1 antibody was used as a loading control. NS: non-stress.

Figure S7

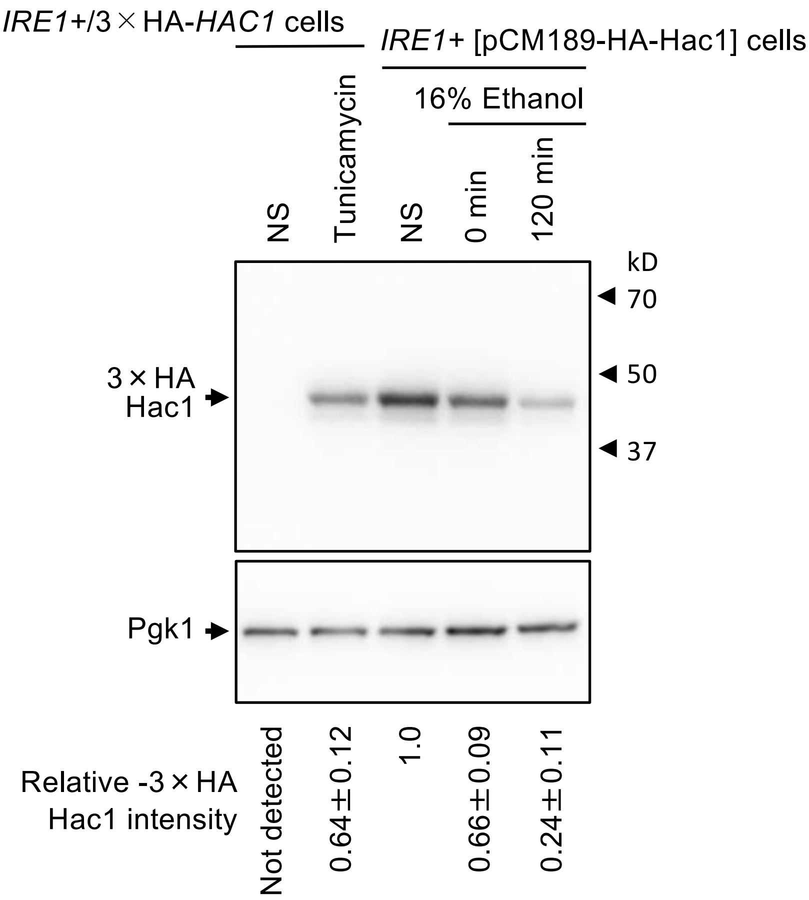

Figure S7 Cellular abundance of Hac1 expressed from the Hac1-expression plasmid
The leftmost and second lanes: *IRE1+*/3×HA-*HAC1* cells (YKY-HA-HAC1), which carry the HA-tagging sequence on the genomic *HAC1* gene, were grown at 30 ° C in SD medium and either stressed with tunicamycin (1 μg/mL, 30 min) or left non-stressed (NS). The third, fourth, and rightmost lanes: *IRE1+* cells (BY4741) carrying the HA epitope-carrying version of pCM189-Hac1 (pCM189-HA-Hac1) were grown at 30 ° C in SD medium and either stressed with ethanol or left non-stressed (NS). Ethanol was added to the cultures using the stepwise addition method, and cells were harvested immediately after the final addition of ethanol to the concentration of 16% (0 min in 16% ethanol) or after an additional 120-min incubation (120 min in 16% ethanol). Crude cell lysates (equivalent to OD_{600}=0.4 cells) were run on standard SDS-PAGE and analyzed by anti-HA western blotting. An anti-Pgk1 antibody was used as an endogenous control.
